# Inheritance and Linkage of Virulence Genes in Chinese Predominant Race CYR32 of the Wheat Stripe Rust Pathogen *Puccinia striiformis* f. sp. *tritici*

**DOI:** 10.3389/fpls.2018.00120

**Published:** 2018-02-08

**Authors:** Long Wang, Dan Zheng, Shuxia Zuo, Xianming Chen, Hua Zhuang, Lili Huang, Zhensheng Kang, Jie Zhao

**Affiliations:** ^1^State Key Laboratory of Crop Stress Biology for Arid Areas and College of Plant Protection, Northwest A&F University, Yangling, China; ^2^Wheat Health, Genetics and Quality Research Unit, United States Department of Agriculture-Agricultural Research Service, Pullman, WA, United States; ^3^Department of Plant Pathology, Washington State University, Pullman, WA, United States; ^4^China–Australia Joint Research Centre for Abiotic and Biotic Stress Management, Northwest A&F University, Yangling, China

**Keywords:** wheat stripe rust, *Puccinia striiformis*, alternate host, sexual reproduction, genetics of virulence

## Abstract

*Puccinia striiformis* f.sp. *tritici* (*Pst*) is the causal agent of stripe (yellow) rust on wheat. It seriously threatens wheat production worldwide. The obligate biotrophic fungus is highly capable of producing new virulent races that can overcome resistance. Studying the inheritance of *Pst* virulence using the classical genetic approach was not possible until the recent discovery of its sexual stage on barberry plants. In the present study, 127 progeny isolates were obtained by selfing a representative Chinese Yellow Rust (CYR) race, CYR32, on *Berberis aggregate*. The parental isolate and progeny isolates were characterized by testing them on 25 wheat lines with different *Yr* genes for resistance and 10 simple sequence repeat (SSR) markers. The 127 progeny isolates were classified into 27 virulence phenotypes (VPs), and 65 multi-locus genotypes (MLGs). All progeny isolates and the parental isolate were avirulent to *Yr5, Yr8, Yr10, Yr15, Yr24, Yr26, Yr32*, and *YrTr1*; but virulent to *Yr1, Yr2, Yr3, Yr4, Yr25, Yr44*, and *Yr76*. The VPs of the parental isolate to nine *Yr* genes (*Yr6, Yr7, Yr9, Yr17, Yr27, Yr28, Yr43, YrA*, and *YrExp2*) and the avirulence phenotype to *YrSP* were found to be heterozygous. Based on the segregation of the virulence/avirulence phenotypes, we found that the VPs to *Yr7, Yr28, Yr43*, and *YrExp2* were controlled by a dominant gene; those to *Yr6, Yr9*, and *YrA* (*Yr73, Yr74*) by two dominant genes; those to *Yr17* and *Yr27* by one dominant and one recessive gene; and the avirulence phenotype to *YrSP* by two complementary dominant genes. Molecular mapping revealed the linkage of 10 virulence/avirulence genes. Comparison of the inheritance modes of the virulence/avirulence genes in this study with previous studies indicated complex interactions between virulence genes in the pathogen and resistance genes in wheat lines. The results are useful for understanding the plant-pathogen interactions and developing wheat cultivars with effective and durable resistance.

## Introduction

Stripe (yellow) rust, caused by *Puccinia striiformis* Westend. f. sp. *tritici* Erikss. (*Pst*), is one of the most destructive diseases of wheat worldwide (Wellings, 2011). Annual loss of wheat grain due to stripe rust was estimated to be about 1 million tons. In China, the most severe epidemics of wheat stripe rust occurred in 1950, 1964, 1990, and 2002 (Chen W. Q. et al., 2009). In 2017, the stripe rust epidemic occurred in 12 provinces, affecting 1.65 million hectares.

The stripe rust fungus is able to produce a large number of races possessing different combinations of virulence genes that are capable of circumventing host resistance genes. Some races can become predominant and widespread due to their virulence spectra, fitness, and aggressiveness (Wan et al., 2017). Among the *Pst* races identified in China using the Chinese set of wheat differentials, CYR32 is one of the most virulent and predominant races. CYR32 was first detected on wheat cultivar Red Abbondanza in Huangzhong, Qinghai Province in 1991 (Wan et al., 2003b). In addition to many other wheat differentials, this race is virulent on wheat cultivar Hybrid 46, which has *Yr4b* and other resistance genes (Wan et al., 2004; Wang and Chen, 2017). More than 80% of Chinese wheat cultivars developed and grown during the 1990s possessed one or more resistance genes from Hybrid 46 (Wan et al., 2007). Race CYR32 rapidly expanded to other wheat-growing areas and quickly became the most predominant race throughout the country (Wan et al., 2003a,b). Eventually, CYR32 reached a maximum frequency of 45.7% in 2001 (Wan et al., 2003a,b) and caused large-scale epidemics in the following years (Li et al., 2016a). To date, CYR32 is still one of the most predominant races in China.

Growing resistant cultivars is a low-cost, effective, and environmental-friendly way to manage stripe rust. However, the pathogen continues producing new races that can overcome race-specific resistance in wheat cultivars (Chen W. Q. et al., 2009). Generally, pathogen variation is attributed to mutation, somatic hybridization and sexual recombination (Little and Manners, 1967; Park and Wellings, 2012; Wang et al., 2016). The previous lack of knowledge on the sexual stage of *Pst* was a huge barrier to understand the role of sexual reproduction in virulence variation. The recent discovery of *Berberis* and *Mahonia* species as alternate hosts of *Pst* (Jin et al., 2010; Wang and Chen, 2013; Zhao et al., 2013) has made it possible to determine the genetics of virulence and map virulence genes through sexual reproduction.

Few studies have been reported on the genetics of *Pst* virulence. Wang et al. (2012) was the first to study the genetics of *Pst* virulence. They obtained 29 progeny isolates by selfing a US isolate of race PST-127 by inoculating young leaves of *Berberis vulgaris* with teliospores. By testing the isolates on *Yr* single-gene lines, they found that the parental isolate was homokaryotic (homozygous) for virulent loci to *Yr1, Yr2*, and *Yr9* and for avirulence loci to *Yr5, Yr15, Yr24, Yr32*, and *YrSP*; whereas its virulence phenotypes (VPs) to *Yr6, Yr7, Yr8, Yr10, Yr17, Yr19, Yr27, Yr43, Yr44, YrExp1, YrExp2, YrTr1*, and *Yr76* (*YrTye*) segregated in different ratios (Wang et al., 2012). Rodriguez-Algaba et al. (2014) selfed an European isolate (DK09/11) on *B. vulgaris* and obtained 16 isolates, which segregated into only three VPs and 11 multi-locus genotypes (MLGs). Due to the fact of largely homozygous parental isolate and a small number of progeny isolates, no attempt was made to determine the genetics of virulence and map virulence loci. Tian et al. (2016) developed 118 isolates by selfing a Chinese *Pst* isolate, Pinglan 17-7, on *Berberis shensiana*. They found that 8 of 24 virulence loci were segregating. The 118 isolates were characterized as 24 VPs based on testing on 24 *Yr* single-gene lines and 82 MLGs using 13 polymorphic simple sequence repeat (SSR) markers. They constructed a preliminary linkage map consisting of eight avirulence/virulence loci and 10 SSR markers. Using the same method, Tian et al. (2017) generated 120 progeny isolates from another Chinese isolate. These progeny isolates were identified as 51 VPs based on virulence testing on 25 *Yr* single-gene lines and 55 MLGs using 11 polymorphic SSR markers. They constructed another linkage map consisting of 4 avirulence loci and 11 SSR markers (Tian et al., 2017). Yuan et al. (2017) generated 119 isolates by selfing a US isolate on barberry and characterized the progeny isolates by virulence phenotyping on 29 wheat lines with single *Yr* genes and genotyping with 173 SSR markers and 10,362 single nucleotide polymorphism (SNP) markers developed from secreted protein genes and genotyping by sequencing. Through cluster analysis and molecular mapping, they determined that *Pst* has six chromosomes in its haploid nucleus and construct the first genetic map of the six chromosomal linkages with 805 markers. They determined that the parental isolate was homozygous for the avirulence loci corresponding to resistance genes *Yr5, Yr10, Yr15, Yr24, Yr32, YrSP, YrTr1, Yr45*, and *Yr53*; homozygous for the virulence locus corresponding to resistance gene *Yr41*; and heterozygous for virulence loci corresponding to 19 single-gene lines. They mapped 17 dominant virulence genes to 2 of the 6 chromosomes.

Under natural conditions, barberry is not infected by *Pst* in the United States (Wang and Chen, 2015; Wang et al., 2015). In China, however, natural *Pst* infection of barberry has been found (Zhao et al., 2013; Li et al., 2016b; Wang et al., 2016). Although at very low frequencies, *Pst* infection on barberry plants may provide opportunities for sexual recombination to produce a large number of races (Zhao et al., 2016).

No matter whether sexual production occurs under natural conditions or not, the genetic state of individual avirulence/virulence loci and interactions between different alleles at the same locus and different loci are the determinants of genetic variations either through sexual recombination, somatic recombination, and/or mutation. The heterozygosity or homozygocity of avirulence/virulence loci and dominance or recessiveness of the opposite phenotypes in a race or population can be used to predict the likelihood of emergence of new virulence genes or races. In turn, this information can be used to predict relative durability of specific resistance genes. This study was aimed to determine the genetics of avirulence and virulence genes by phenotyping and genotyping a *Pst* progeny population derived from CYR32, a predominant race in China, through selfing on *B. aggregate*.

## Materials and methods

**Figure 1:**
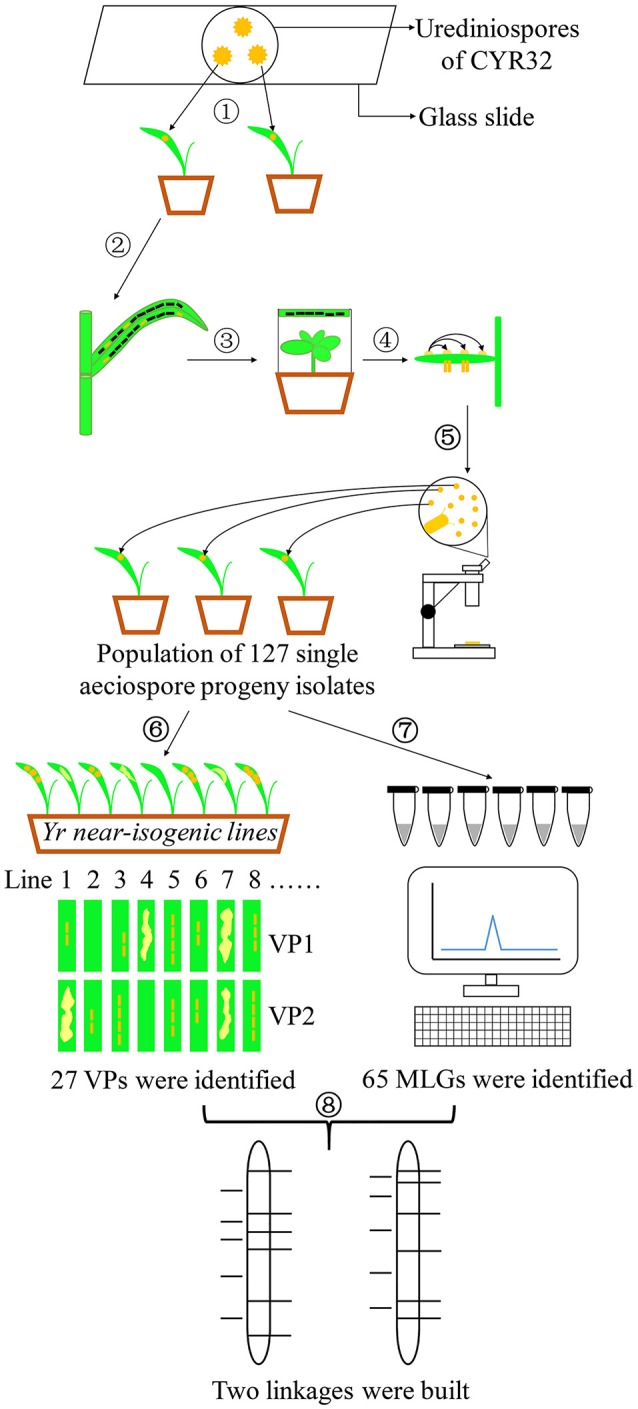
Experimental flow chart. (1) Obtaining a single-urediniospore isolate of CYR32. (2) Producing teliospores. (3) Inoculating barberry plants with basidiospores produced from germinating teliospores. (4) Fertilization by transferring pycniospores in nectar from a pycium to another. (5) Developing a segregating population consisting of 127 uredinial isolates from individual aeciospores. (6) Phenotype test of the 127 progeny isolates on 25 *Yr* single-gene lines. (7) DNA extraction and genotype detection using SSR markers. (8) Linkage construction.

Figure 1 illustrates the experimental procedures, including selection of *Pst* isolate and teliospore production; inoculation of barberry and aeciospore production; development, phenotyping and genotyping of a progeny uredinial population; and linkage construction.

### Single-urediniospore isolation and teliospore production

Leaf samples of *Pst* were collected from a wheat field in Tianshui, Gansu Province in 2014, purified by picking a single uredinium following the method described by Wan et al. (2004). The VP of each single-uredinium isolate was determined using a set of differentials consisting of 19 wheat genotypes used to differentiate Chinese *Pst* races (Wan et al., 2004). An isolate identified as race CYR32 was selected and used to generate single-urediniospore isolates. Urediniospores of single-urediniospore isolates were propagated on wheat cultivar (cv.) Mingxian 169 that is susceptible to all known Chinese *Pst* races. Each single-uediniospore isolate was tested again on the same set of differentials to verify the VP and one isolate was selected for producing teliospores and included in the phenotyping and genotyping tests.

For teliospore production, adult plants of Mingxian 169 were inoculated with urediniospores of the selected isolate to represent CYR32 and the inoculated plants were incubated in a dew chamber with 100% relative humidity at 10°C in dark for 24 h. After incubation, plants were transferred to a growth chamber with a photoperiod of 16 h at 16°C and 8 h darkness at 13°C for urediniospore production. At the emergence of uredinia, daytime temperature was raised to 25°C for inducing telial formation. Wheat leaves bearing black telia were harvested, dried for 3 days at room temperature, and kept in a desiccator at 4°C for later use.

### Inoculation of *B. aggregate* and aeciospore production

Young plants of *B. aggregate* were collected from Tianshui and grown in small pots. For inoculation, each small plant bearing fresh leaves was placed in an open-top plastic cylinder containing water to keep high moisture. Before inoculating barberry leaves, wheat leaves bearing teliospores were immersed in distilled water overnight at room temperature and then put onto 2% (w/v) water agar in a petri dish. When teliospores began to produce basidiospores, the petri dish was turned over and placed on the top of the plastic cylinder containing a *B. aggregate* plant. Inoculated barberry plants were kept in a dew chamber with 100% relative humidity and a light period of 12 h at 16°C and 12 h darkness at 12°C for 3–4 days. Plants were then transferred to a growth chamber with the same temperature conditions as for urediniospore production described the above.

About 7 days after inoculation when pycnia were observed on the adaxial surface of a barberry leaf and formed obvious nectars (or honeydews) on pycnial ostiles, the nectar from one pycnium was transferred to another pycnium using a clean wheat leaf tip for fertilization. More than 200 transfers were conducted to get an adequate number of aecia. When aecia were observed on the baxial surface of the barberry leaves about 7 days later, the plastic cylinder was removed to avoid releasing aeciospores due to high moisture.

### Development of a sexual population

When aecia reached approximately 3–5 mm in length, an aecium was excised and placed onto a clean glass slide and broken gently to release aeciospores. An aeciospore was transferred to a leaf of l8-day-old seedling of Mingxian 169 with an eyelash-made picker under a dissect microscope, One leaf was inoculated with only one aeciospore, and more than 200 leaves were inoculated to producing an adequate number of aeciospore-derived urediniospore isolates. Inoculated plants were placed in a dew chamber and kept at 10°C and 100% relative humidity for 24 h in dark. Plants were then transferred to a growth chamber with temperatures set at 16°C at daytime and 13°C at nighttime and 16 h light and 8 h dark. About 12 days after inoculation and before sporulation, plants were covered with a plastic cylinder to prevent cross contamination. Urediniospores produced from each aeciospore inoculation were collected by shaking into a glass test tube and stored in a desiccator at 4°C.

### Virulence tests

A set of 25 wheat genotypes (Supplementary Table 1) was used to determine the VPs of the progeny isolates, and Mingxian 169 was used as a susceptible check. Seedlings of the wheat genotypes were inoculated using the method described by Zhao et al. (2013). After inoculation, plants were moved to a growth chamber with the same conditions as described above. Infection types (ITs) were recorded 18–20 days after inoculation using the 0–9 scale described by Line and Qayoum (1992). The virulence test was repeated twice. According to Wan and Chen (2014), ITs 0–6 were considered as avirulent (A) and 7–9 as virulent (V).

### DNA extraction

Genomic DNA was extracted from urediniospores following the method described by Aljanabi and Martinez (1997) with modifications. Approximately 50 mg urediniospores of an isolate were transferred into a 2 mL clean centrifuge tube with steel beads (4 mm in diameter), ground for 6 min to fine powder in a swing mill (TissueLyser II, QIAGEN, Hilden, Germany), and added with 500 μL of pre-heated CTAB extraction buffer (0.4 M NaCl; 10 mM Tris-HCl, pH 8; 2 mM EDTA, pH 8, 65°C). The mixture was blended gently, incubated at 65°C for 1 h, with gentle tipping every 10 min, added with 750 μL phenol-chloroform-isoamylalcohol solution (v:v:v = 25:24:1, pH 7.8) and centrifuged at 10,000 g for 10 min at 4°C. After the upper phase was transferred to a clean tube and added with 300 μL chloroform, the mixture was centrifuged at 10,000 g for 10 min at 4°C. The upper phase was transferred to a new tube, added with pre-cooled 800 μL isopropanol and kept at −20°C overnight. After centrifuging at 10,000 g for 30 min at 4°C and discarding the supernatant, the DNA pellet was washed first with 75% (v/v) and then 100% ethanol. After drying for 2 h at room temperature, the DNA pellet was dissolved in 50 μL of 1 × TE buffer (10 mM Tris-HCl and 1 mM EDTA, pH 8.0). The solution was added with 1.0 mL 20 μg/mL RNase and incubated at 37°C for 30 min to digest RNA. DNA was re-precipitated and dissolved in TE buffer as described above. DNA concentration was measured using an ND-1000 spectrophotometer (Bio-Rad Laboratories, Hercules, CA, USA) and diluted to 50 ng/μL with 1X TE buffer to be used as template in polymerase chain reaction (PCR).

### SSR genotyping

To genotype the parental and progeny isolates, 150 pairs of *Pst* SSR primers (Enjalbert et al., 2002; Bahri et al., 2008; Chen C. Q. et al., 2009; Cheng et al., 2012; Bailey et al., 2013; Luo et al., 2015) were synthesized and labeled with fluorescence at 5′-end (Shanghai Sangon Bio-tech Company, Shanghai, China). PCR reactions were composed of 12.5 μL of 2x *Taq* PCR Master Mix (*Taq* DNA polymerase: 0.05 U/μL; MgCl_2_, 4 mM; dNTPs, 0.4 mM), 1.0 μL of each of primers (10 mM), 2.0 μL of DNA, and 8.5 μL ddH_2_O. PCR was performed with touch-down in an S1000 Thermal Cycler (Bio-Rad Laboratories). The PCR program was set up as follows: 95°C for 5 min; 10 cycles of 94°C for 45 s, 64°C for 45 s (with a reduction of 1°C at each cycle) and 72°C for 45 s; followed by 25 cycles with 94°C for 45 s, 54°C for 45 s, and 72°C for 45 s; and one final extension at 72°C for 10 min. PCR products were analyzed using a 3730XL DNA Analyzer (Applied Biosystems, Waltham, MA, USA). SSR amplicons were scored using software GeneMarker HID (Holland and Parson, 2011).

### Data analysis

Virulence phenotypes of the progeny isolates were determined using software VAT 1.0 (Kosman and Leonard, 2007). Chi-squared test (χ^2^) was used to determine the goodness of fit for the observed segregations of virulent verses avirulent phenotypes and SSR alleles to the expected ratio of a single locus. The correlation coefficient (*R*^2^) between VPs and MLGs was determined using software NTSYS-pc (ver. 2.10) MXCOMP. A genetic linkage map was constructed using software GACD (http://www.isbreeding.net/). The threshold of the logarithm of odd (LOD) score was set as 2.50 after a permutation test.

## Results

### Virulence phenotypes

A total of 127 single-spore isolates were obtained through sexual reproduction on barberry plants by selfing a race CYR32 isolate. The progeny isolates were all avirulent to eight wheat lines (*Yr5, Yr8, Yr10, Yr15, Yr24, Yr26, Yr32*, and *YrTr1*) and virulent to seven lines (*Yr1, Yr2, Yr3, Yr4, Yr25, Yr44*, and *Yr76* (*YrTye*) but segregated on 10 lines (*Yr6, Yr7, Yr9, Yr17, Yr27, Yr28, Yr43, YrA, YrExp2*, and *YrSP*). The results indicated that the CYR32 isolate was homozygous avirulent for the first group of eight avirulence loci, homozygous virulent for the second group of seven virulence loci, and heterozygous for the third group of 10 loci. Based on the different reactions on the third group of 10 segregating loci, the 127 progeny isolates were identified as 27 VPs (Table 1, Supplementary Table 2).

**Table 1:** Virulence phenotypes (VPs) of CYR32 and its progeny isolates on wheat *Yr* gene lines.

**VP**	**No. of isolates**	**Avirulence (A) and virulence (V) on** ***Yr*** **single-gene line^a^**									
		***Yr6***	***Yr7***	***Yr9***	***Yr17***	***Yr27***	***Yr28***	***Yr43***	***YrA***	***YrExp2***	***YrSP***
CYR32	NA^b^	V	V	V	V	V	V	V	V	V	A
1	33	V	V	V	V	V	V	V	V	V	A
2	42	V	V	V	V	V	V	V	V	V	V
3	3	V	V	V	V	V	V	A	V	V	V
4	1	V	A	V	V	V	V	V	V	A	V
5	1	V	A	V	V	V	V	V	V	V	A
6	1	V	V	V	A	V	V	V	V	V	A
7	6	V	V	V	V	V	A	V	V	V	A
8	2	V	V	V	V	V	V	A	V	V	A
9	3	V	V	V	V	V	V	V	V	A	A
10	2	V	A	V	V	V	V	A	V	A	V
11	4	V	A	V	V	V	V	A	V	V	A
12	1	V	A	V	V	V	V	V	V	A	A
13	1	V	V	A	V	V	V	V	V	A	A
14	1	V	V	V	V	A	A	V	V	A	V
15	1	V	V	V	V	V	A	A	V	A	V
16	4	V	V	V	V	V	V	A	V	A	A
17	3	V	A	V	V	V	V	A	V	A	A
18	2	V	A	V	V	V	A	A	V	A	A
19	1	V	V	V	V	A	A	A	V	A	A
20	1	V	A	V	A	V	A	A	V	A	A
21	1	V	A	A	A	A	A	A	V	A	V
22	1	V	A	V	A	V	A	A	A	A	A
23	1	V	V	A	A	A	A	A	A	A	V
24	2	V	A	A	A	A	A	A	V	A	A
25	1	V	A	V	A	A	A	A	A	A	A
26	1	A	A	A	A	A	A	V	A	A	A
27	7	A	A	A	A	A	A	A	A	A	A

a *The parental isolate CYR32 and the progeny isolates were tested on 25 Yr single-gene lines. CYR32 and all progeny isolates were avirulent (infection types 0–6) to Yr5, Yr8, Yr10, Yr15, Yr24, Yr26, Yr32, and YrTr1, but virulent (infection types 7–9) to Yr1, Yr2, Yr3, Yr4, Yr25, Yr44, and Yr76 (YrTye). CYR32 was the same as VP 1*.

b *NA, not applicable*.

VP1 had 33 isolates with the same VP as CYR32, virulent to all of the 10 *Yr* genes lines in the third group except *YrSP* (Table 1). With 42 isolates, VP2 was the largest VP having virulence to *YrSP* in contrast to the avirulence in the parental isolate. Compared to the parental isolate CYR32, VP3 changed from virulence to avirulence to *Yr43* and from avirulence to virulence to *YrSP*. The virulence spectra of the 49 isolates in 24 VPs (VP4–VP27) had relative narrow virulence spectra compared to the parental isolate, including VP27 that was avirulent to all of the 10 *Yr* gene lines producing segregated phenotypes (Table 1). These results indicate that a *Pst* isolate with several heterozygous virulence/avirulence loci can sexually produce a large number of races, which can be either more virulent or less virulent than the original isolate.

### Inheritance of virulence/avirulence

Different segregation ratios were obtained for the heterozygous virulence/avirulence loci. Each of the avirulence phenotypes to *Yr7, Yr28, Yr43*, and *YrExp2* in the parental isolate was found to be controlled by a single recessive gene or the VPs were controlled by a dominant gene, indicated by the 1:3 avirulent to virulent ratio (Table 2). Two loci were found to be involved in the virulence/avirulence phenotypes to the other six *Yr* genes. A 1:15 ratio for avirulence to virulence was obtained when the progeny isolates were on the wheat lines with *Yr6, Yr9*, and *YrA* (*Yr73* and *Yr74*), indicating two complementary recessive genes for the avirulence phenotypes. A 3 avirulent:13 virulent ratio was obtained for the segregations on the wheat lines with *Yr17* or *Yr27*, indicating that the parental isolate has a dominant gene and a recessive gene in complementary for the VPs to these resistance genes. For the 10 segregating loci, the parental isolate was avirulent only to *YrSP*. The progeny isolates segregated at a 9:7 ratio for avirulence (A):virulence (V), indicating that the parental isolate has two complementary dominant genes for the *YrSP* avirulence phenotype.

**Table 2:** Infection types, segregation of avirulence/virulence in the selfed progeny isolates derived from *Puccinia striiformis* f. sp. *tritici* race CYR32 on *Yr* single-gene lines and probability (*P*) values based on Chi-squared tests.

**Wheat line with *Yr* gene**	**Infection type of CYR32**	**Observed number of progeny isolates**		**Expected ratio (A:V)**	***P***
		**Avirulence**	**Virulence**		
*Yr7*	9	28	99	1:3	0.44
*Yr28*	9	26	101	1:3	0.24
*Yr43*	9	36	91	1:3	0.38
*YrExp2*	9	35	92	1:3	0.51
*Yr6*	9	8	119	1:15	0.98
*Yr9*	9	13	114	1:15	0.06
*YrA* (*Yr73, Yr74*)^a^	9	11	116	1:15	0.26
*Yr17*	9	16	111	3:13	0.08
*Yr27*	9	15	112	3:13	0.05
*YrSP*	1	75	52	9:7	0.52

a *Referred to Dracatos et al. (2016)*.

### SSR markers and linkage map

Of the 150 SSR markers screened, 10 produced co-dominant amplicons in the parental isolate and segregated in the progeny population. As an example, the different amplicons of the marker scaffold 962-172974 locus are shown in Supplementary Figure 1. As expected, the correlation coefficient between the virulent phenotype and SSR genotype data was very low (*R*^2^ = 0.07).

**Figure 2:**
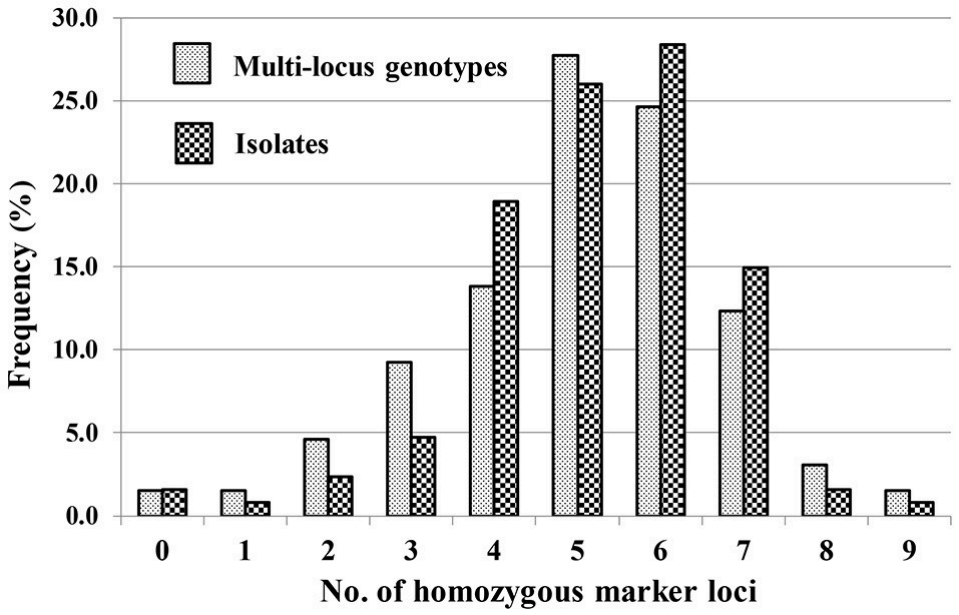
Frequencies of progeny isolates with different numbers of homozygous SSR marker loci for the 10 loci at which the parental isolate was heterozygous.

A total of 65 MLGs were identified from the 127 progeny isolates (Table 3, Supplementary Table 3). The first MLG (MLG 1), identified from two progeny isolates, was the same as the parental isolate with the heterozygosity at all of the 10 SSR marker loci. The other 64 MLG differed from the parental isolate by different numbers (1–9) of homozygous marker loci. The frequency distribution of the classes based on the number of marker loci becoming homozygous was close to a normal distribution based on either MLGs or isolates (Figure 2), indicating that the progeny population was randomly generated and the segregating markers were suitable for constructing a linkage map.

**Table 3:** Multi-locus genotypes (MLG) of the parental isolate CYR32 and progeny isolates determined based on 10 heterozygous SSR loci.

**MLG**	**No. of isolates**	**No. of homozygous loci**	**Amplified fragment size (base pairs) of SSR markers^a^**																			
			**M1**		**M2**		**M3**		**M4**		**M5**		**M6**		**M7**		**M8**		**M9**		**M10**	
			**a1**	**a2**	**a1**	**a2**	**a1**	**a2**	**a1**	**a2**	**a1**	**a2**	**a1**	**a2**	**a1**	**a2**	**a1**	**a2**	**a1**	**a2**	**a1**	**a2**
**CYR32**	**NA^b^**	**0**	**315**	**318**	**286**	**298**	**237**	**253**	**274**	**288**	**211**	**214**	**224**	**233**	**231**	**253**	**357**	**365**	**339**	**349**	**308**	**323**
1	2	0	315	318	286	298	237	253	274	288	211	214	224	233	231	253	357	365	339	349	308	323
2	1	1	315	318	286	298	237	253	*274*	*274*	211	214	224	233	231	253	357	365	339	349	308	323
3	1	2	315	318	286	298	237	253	*274*	*274*	*214*	*214*	224	233	231	253	357	365	339	349	308	323
4	1	2	315	318	286	298	237	253	274	288	211	214	224	233	231	253	357	365	*339*	*339*	*308*	*308*
5	1	2	315	318	286	298	237	253	*274*	*274*	211	214	*233*	*233*	231	253	357	365	339	349	308	323
6	1	3	*318*	*318*	*286*	*286*	*253*	*253*	274	288	211	214	224	233	231	253	357	365	339	349	308	323
7	1	3	*315*	*315*	286	298	*253*	*253*	274	288	211	214	224	233	*253*	*253*	357	365	339	349	308	323
8	1	3	*315*	*315*	286	298	*253*	*253*	274	288	211	214	*233*	*233*	231	253	357	365	339	349	308	323
9	1	3	*318*	*318*	*286*	*286*	237	253	*274*	*274*	211	214	224	233	231	253	357	365	339	349	308	323
10	1	3	315	318	*286*	*286*	237	253	274	288	*214*	*214*	224	233	*253*	*253*	357	365	339	349	308	323
11	1	3	315	318	286	298	237	253	274	288	211	214	*224*	*224*	231	253	357	365	*339*	*339*	*308*	*308*
12	1	4	315	318	286	298	237	253	274	288	*214*	*214*	*224*	*224*	*231*	*231*	*365*	*365*	339	349	308	323
13	1	4	315	318	286	298	237	253	274	288	*214*	*214*	*233*	*233*	*253*	*253*	*357*	*357*	339	349	308	323
14	1	4	315	318	286	298	*237*	*237*	274	288	211	214	*224*	*224*	*231*	*231*	*365*	*365*	339	349	308	323
15	1	4	315	318	*298*	*298*	237	253	274	288	211	214	*NB^c^*	*NB*	*253*	*253*	357	365	*339*	*339*	308	323
16	3	4	*318*	*318*	286	298	237	253	274	288	211	214	224	233	*253*	*253*	*365*	*365*	339	349	*323*	*323*
17	1	4	*318*	*318*	286	298	237	253	274	288	211	214	*224*	*224*	*253*	*253*	*365*	*365*	339	349	308	323
18	1	4	*315*	*315*	286	298	237	253	274	288	*214*	*214*	*224*	*224*	*253*	*253*	357	365	339	349	308	323
19	14	4	*318*	*318*	*286*	*286*	*253*	*253*	274	288	*211*	*211*	224	233	231	253	357	365	339	349	308	323
20	1	4	*318*	*318*	*298*	*298*	237	253	274	288	*211*	*211*	*233*	*233*	231	253	357	365	339	349	308	323
21	1	5	*315*	*315*	*298*	*298*	237	253	274	288	*214*	*214*	224	233	*231*	*231*	*357*	*357*	339	349	308	323
22	1	5	*315*	*315*	286	298	237	253	*274*	*274*	*214*	*214*	224	233	231	253	*365*	*365*	339	349	*308*	*308*
23	1	5	*315*	*315*	286	298	*237*	*237*	*274*	*274*	*214*	*214*	224	233	231	253	*365*	*365*	339	349	308	323
24	1	5	*315*	*315*	286	298	*253*	*253*	*288*	*288*	*214*	*214*	*233*	*233*	231	253	357	365	339	349	308	323
25	3	5	*318*	*318*	*286*	*286*	*253*	*253*	274	288	*211*	*211*	*224*	*224*	231	253	357	365	339	349	308	323
26	1	5	*318*	*318*	286	298	237	253	*288*	*288*	211	214	224	233	*253*	*253*	357	365	*339*	*339*	*308*	*308*
27	1	5	*318*	*318*	286	298	237	253	274	288	*211*	*211*	*NB*	*NB*	231	253	*357*	*357*	339	349	*308*	*308*
28	1	5	*318*	*318*	286	298	237	253	274	288	211	214	224	233	*231*	*231*	*365*	*365*	*339*	*339*	*308*	*308*
29	7	5	*318*	*318*	286	298	237	253	274	288	211	214	*224*	*224*	*253*	*253*	*365*	*365*	339	349	*323*	*323*
30	1	5	315	318	*286*	*286*	*237*	*237*	*274*	*274*	*211*	*211*	224	233	*231*	*231*	357	365	339	349	308	323
31	1	5	315	318	*286*	*286*	237	253	274	288	211	214	*233*	*233*	*253*	*253*	357	365	*339*	*339*	*NB*	*NB*
32	1	5	315	318	*298*	*298*	237	253	*274*	*274*	*214*	*214*	*224*	*224*	231	253	357	365	339	349	*323*	*323*
33	2	5	315	318	*298*	*298*	*253*	*253*	274	288	*211*	*214*	*233*	*233*	*253*	*253*	357	365	*339*	*339*	308	323
34	1	5	315	318	286	298	237	253	*274*	*274*	*211*	*211*	*233*	*233*	*231*	*231*	*357*	*357*	339	349	308	323
35	1	5	315	318	286	298	*237*	*237*	*288*	*288*	*211*	*211*	*224*	*224*	231	253	*357*	*357*	339	349	308	323
36	4	5	315	318	286	298	237	253	*288*	*288*	211	214	*224*	*224*	231	253	*357*	*357*	*339*	*339*	*308*	*308*
37	4	5	315	318	286	298	*253*	*253*	*288*	*288*	211	214	224	233	231	253	*357*	*357*	*349*	*349*	*323*	*323*
38	1	5	315	318	286	298	*253*	*253*	274	288	211	214	224	233	*253*	*253*	*357*	*357*	*349*	*349*	*323*	*323*
39	1	6	315	318	286	298	*253*	*253*	*288*	*288*	211	214	*224*	*224*	231	253	*357*	*357*	*349*	*349*	*323*	*323*
40	9	6	315	318	286	298	*253*	*253*	*288*	*288*	211	214	*233*	*233*	231	253	*357*	*357*	*349*	*349*	*323*	*323*
41	1	6	315	318	286	298	237	253	*288*	*288*	*214*	*214*	224	233	*231*	*231*	*365*	*365*	*339*	*339*	*308*	*308*
42	1	6	315	318	286	298	*253*	*253*	*274*	*274*	211	214	224	233	*253*	*253*	*357*	*357*	*349*	*349*	*323*	*323*
43	2	6	315	318	286	298	*237*	*237*	*274*	*274*	*214*	*214*	*224*	*224*	231	253	357	365	*349*	*349*	*323*	*323*
44	1	6	315	318	*286*	*286*	*253*	*253*	274	288	*214*	*214*	*233*	*233*	231	253	357	365	*339*	*339*	*308*	*308*
45	1	6	315	318	*286*	*286*	*253*	*253*	274	288	211	214	224	233	*231*	*231*	*357*	*357*	*349*	*349*	*323*	*323*
46	5	6	315	318	*286*	*286*	*253*	*253*	274	288	*211*	*211*	224	233	231	253	*357*	*357*	*349*	*349*	*323*	*323*
47	2	6	*318*	*318*	286	298	237	253	*274*	*274*	211	214	224	233	*231*	*231*	*365*	*365*	*339*	*339*	*308*	*308*
48	1	6	*318*	*318*	*298*	*298*	237	253	274	288	*211*	*211*	*233*	*233*	231	253	*357*	*357*	339	349	*308*	*308*
49	3	6	*315*	*315*	286	298	*237*	*237*	*274*	*274*	*214*	*214*	*233*	*233*	231	253	*365*	*365*	339	349	308	323
50	1	6	*315*	*315*	286	298	237	253	*274*	*274*	*214*	*214*	*224*	*224*	231	253	*365*	*365*	339	349	*308*	*308*
51	5	6	*315*	*315*	286	298	237	253	*274*	*274*	*214*	*214*	*233*	*233*	231	253	*365*	*365*	339	349	*308*	*308*
52	1	6	*315*	*315*	*286*	*286*	237	253	*288*	*288*	*214*	*214*	*233*	*233*	231	253	*357*	*357*	339	349	308	323
53	1	6	*315*	*315*	*298*	*298*	237	253	*274*	*274*	*214*	*214*	*NB*	*NB*	231	253	357	365	339	349	*323*	*323*
54	1	6	*315*	*315*	*298*	*298*	*253*	*253*	*288*	*288*	211	214	*233*	*233*	*253*	*253*	357	365	339	349	308	323
55	1	7	315	318	*298*	*298*	237	253	*288*	*288*	*211*	*211*	*224*	*224*	231	253	*357*	*357*	*339*	*339*	*308*	*308*
56	1	7	315	318	*286*	*286*	*253*	*253*	*288*	*288*	*211*	*211*	224	233	231	253	*357*	*357*	*349*	*349*	*323*	*323*
57	1	7	315	318	*286*	*286*	237	253	*288*	*288*	*214*	*214*	224	233	*231*	*231*	*365*	*365*	*339*	*339*	*308*	*308*
58	12	7	315	318	*286*	*286*	*253*	*253*	274	288	*211*	*211*	*224*	*224*	231	253	*357*	*357*	*349*	*349*	*323*	*323*
59	1	7	315	318	*286*	*286*	*253*	*253*	274	288	*211*	*211*	*233*	*233*	231	253	*357*	*357*	*349*	*349*	*323*	*323*
60	1	7	*318*	*318*	*NB*	*NB*	237	253	274	288	*211*	*211*	*224*	*224*	231	253	*NB*	*NB*	*339*	*339*	*NB*	*NB*
61	1	7	*318*	*318*	*286*	*286*	*253*	*253*	274	288	*211*	*211*	*233*	*233*	*231*	*231*	357	365	339	349	*323*	*323*
62	1	7	*318*	*318*	286	298	*237*	*237*	*288*	*288*	*214*	*214*	224	233	*231*	*231*	*357*	*357*	*339*	*339*	308	323
63	1	8	*318*	*318*	*298*	*298*	237	253	*288*	*288*	*214*	*214*	224	233	*231*	*231*	*357*	*357*	*349*	*349*	*323*	*323*
64	1	8	315	318	*286*	*286*	237	253	288	288	*214*	*214*	*224*	*224*	*231*	*231*	*365*	*365*	*339*	*339*	*308*	*308*
65	1	9	*NB*	*NB*	*286*	*286*	*253*	*253*	*NB*	*NB*	211	214	*224*	*224*	*253*	*253*	*NB*	*NB*	*349*	*349*	*NB*	*NB*

a *The 10 SSR markers used are: M1 = RJ6N, M2 = RJ24, M3 = scaffold 821-34567, M4 = scaffold 750-153307, M5 = scaffold 962-172974, M6 = CPS09, M7 = SUNIPst 09-48, M8 = SUNIPst 10-06, M9 = SUNIPst 11-21, M10 = SUNIPst 15-30. Sizes of amplified fragments are given in base pairs. a1 = allele 1, and a2 = allele 2. MLG = multi-locus genotype*.

b *NA = not applicable*.

c *NB = no band*.

**Figure 3:**
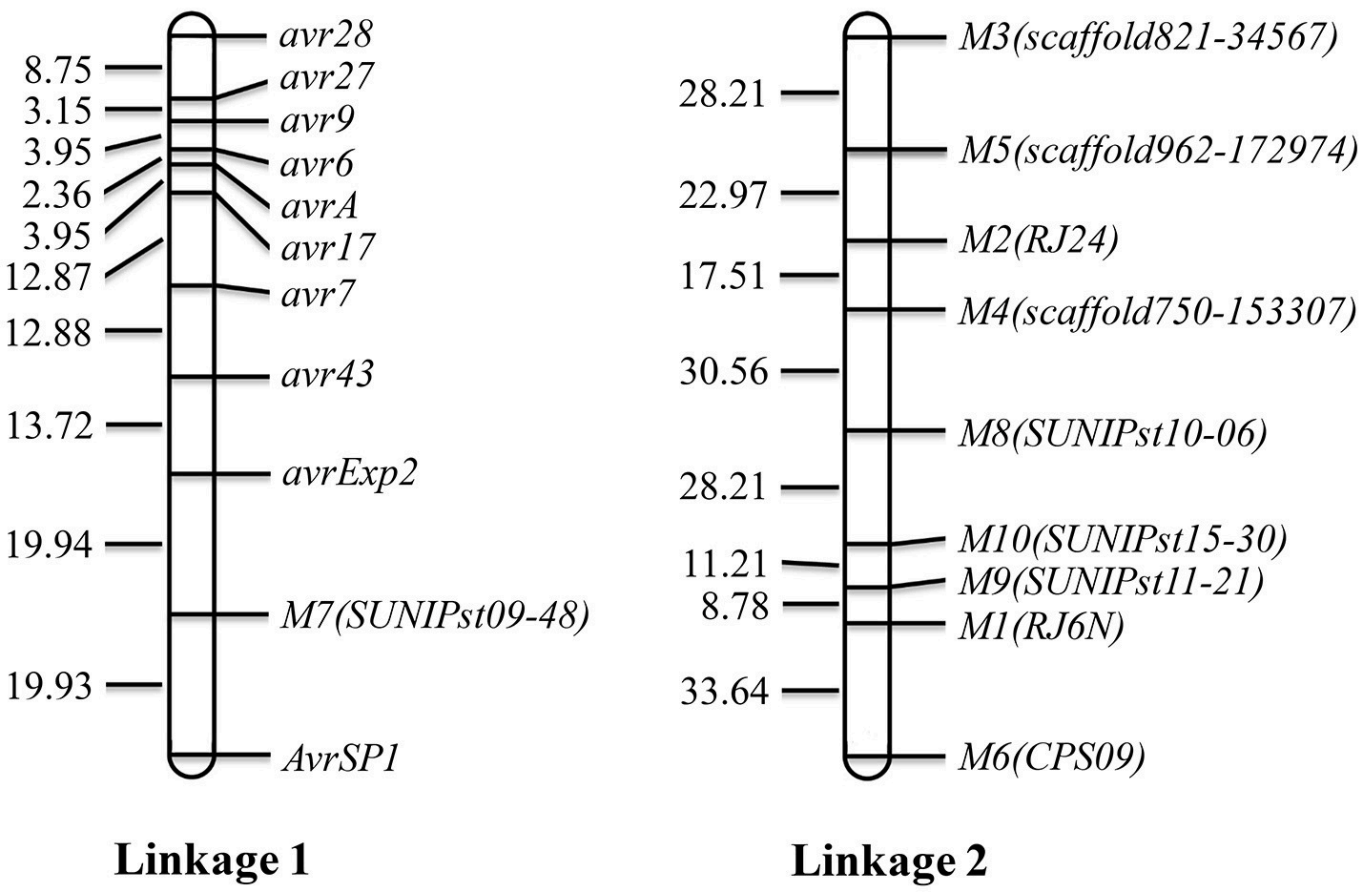
Linkage map constructed with avirulence genes and SSR markers. *Avr* denotes a dominant avirulence gene and *avr* denotes a recessive avirulence gene.

Using the 10 segregating SSR markers and the 10 VPs, a map consisting of two linkages was constructed (Figure 3). Linkage 1 consisted of 10 virulence/avirulence genes and 1 SSR marker and linkage 2 consisted of nine SSR markers. The genetic distances between two neighboring virulence loci ranged from 2.36 between *avr6* and *avrA* to 39.87 cM between *avrExp2* and *AvrSP1*.

## Discussion

### Homozygous and heterozygous virulence loci in the parental isolates

In this study, we generated a sexual population by selfing an isolate of predominant Chinese *Pst* race CYR32 through sexual reproduction on barberry plants. By testing the parental and progeny isolates on 25 wheat lines possessing different *Yr* genes for resistance to stripe rust, we discovered that the parental isolate is homozygous for its avirulence phenotypes to *Yr5, Yr8, Yr10, Yr15, Yr24, Yr26, Yr32*, and *YrTr1*; homozygous for its VPs to *Yr1, Yr2, Yr3, Yr4, Yr25, Yr44*, and *Yr76* (*YrTye*); and heterozygous for its VPs to *Yr6, Yr7, Yr9, Yr17, Yr27, Yr28, Yr43, YrA* (*Yr73, Yr74*), and *YrExp2*; and avirulence to *YrSP*. Homozygous or heterozygous virulence/avirulence phenotypes in different selfed *Pst* populations have been previously reported by Tian et al. (2016, 2017) using isolates representing two different Chinese races and by Yuan et al. (2017) for a US race (Table 4).

**Table 4:** Comparisons of the CYR32 progeny population with previously reported sexual populations in virulence phenotypes and ratios on *Yr* gene lines.

	**Avirulence: Virulence ratio^b^**							
	**CYR32**		**Pinglan 17-7**		**GS-2013**		**PSTv-11**	
***Yr* gene line^a^**	**Obs**.	**Exp**.	**Obs**.	**Exp**.	**Obs**.	**Exp**.	**Obs**.	**Exp**.
*Yr1*	0:127	0:1	0:82	0:1	21:99	1:3	31:88	1:3
*Yr2*	0:127	0:1	0:82	0:1	40:80	7:9	65:54	7:9
*Yr4*	0:127	0:1	5:77	1:15	115:5	15:1	ND	ND
*Yr25*	0:127	0:1	0:82	0:1	0:120	0:1	34:85	1:3
*Yr44*	0:127	0:1	3:79	1:15	15:105	3:13	5:114	1:15
*Yr76*	0:127	0:1	ND	ND	20:100	3:13	8:111	1:15
*Yr5*	127:0	1:0	82:0	1:0	120:0	1:0	119:0	1:0
*Yr8*	127:0	1:0	82:0	1:0	90:30	3:1	36:83	1:3
*Yr10*	127:0	1:0	0:82	0:1	120:0	1:0	119:0	1:0
*Yr15*	127:0	1:0	82:0	1:0	120:0	1:0	119:0	1:0
*Yr24* (=*Yr26*)	127:0	1:0	0:82	0:1	120:0	1:0	119:0	1:0
*Yr26* (=*Yr24*)	127:0	1:0	0:82	0:1	120:0	1:0	ND	ND
*Yr32*	127:0	1:0	7:75	1:15	115:5	15:1	119:0	1:0
*YrTr1*	127:0	1:0	82:0	1:0	112:8	15:1	119:0	1:0
*Yr7*	28:99	1:3	0:82	0:1	92:28	3:1	31:88	1:3
*Yr28*	26:101	1:3	16:66	1:3	19:101	3:13	35:84	1:3
*Yr43*	36:91	1:3	65:17	3:1	100:20	13:3	5:114	1:15
*YrExp2*	35:92	1:3	0:82	0:1	94:26	3:1	46:73	7:9
*Yr6*	8:119	1:15	58:24	3:1	6:114	1:15	25:94	1:3
*Yr9*	13:114	1:15	0:82	0:1	20:100	3:13	30:89	1:3
*YrA* (*Yr73, Yr74*)	11:116	1:15	0:82	0:1	0:120	0:1	29:90	1:3
*Yr17*	16:111	3:13	0:82	0:1	0:120	0:1	56:63	7:9
*Yr27*	15:112	3:13	13:69	1:3	22:98	1:3	29:90	1:3
*YrSP*	75:52	9:7	69:13	3:1	94:26	3:1	119:0	1:0

a *Yr gene lines are in the Avocet S background with Yr73 (Yuan et al., 2017), except Yr4 that is in Hybrid 46 with additional genes and YrA (Yr73+Yr74) (Wan and Chen, 2014; Dracatos et al., 2016)*.

b *The data of the Pinglan 17-7, GS-2013, and PSTv-11 selfed populations were from Tian et al. (2016), Tian et al. (2017), and Yuan et al. (2017), respectively. Obs., observed ratio; and Exp., expected ratio*.

*ND = no data*.

Homozygous avirulence phenotypes to both *Yr5* and *Yr15* were identified in the present study and also in the previous studies (Tian et al., 2016, 2017; Yuan et al., 2017), indicating that the effectiveness of these resistance genes in both China and the US. A resistance gene is relatively durable when their corresponding avirulence gene is homozygous in the pathogen population compared to a resistance gene with heterozygous avirulence gene, because two steps of mutations are needed to convert avirulence to virulence if avirulence is dominant (Yuan et al., 2017). In addition to the avirulence phenotypes to *Yr5* and *Yr15*, avirulence genes to six other *Yr* genes (*Yr8, Yr10, Yr24, Yr26, Yr32*, and *YrTr1*) were also homozygous in the present study. The homozygous avirulence to *Yr8* was also found in the population derived from another Chinese isolate, Pinglan 17-7 (Tian et al., 2016).

### The number of loci controlling virulence phenotypes

In the present study, the segregations of virulence or avirulence phenotypes on 10 *Yr*-gene lines allowed us to determine the number of genes controlling the phenotypes. The VPs to four resistance genes (*Yr7, Yr28, Yr43*, and *YrExp2*) were each controlled by a single locus, those on five *Yr*-gene near-isogenic lines (*Yr6, Yr9, YrA, Yr17*, and *Yr27*) were controlled by two loci, and the avirulence to *YrSP* was also controlled by two loci (Table 4). Both single-locus and two-loci controlled VPs were also reported in the previous studies. In the study of the selfed population derived from Chinese isolate Pinglan 17-7, Tian et al. (2016) reported one locus controlling the VPs on wheat lines possessing *Yr6, Yr27, Yr28, Yr43*, and *YrSP* and two loci controlling the VPs on lines *Yr4, Yr32*, and *Yr44* (Table 4). In the study of the GS-2013 population (because the isolate did not have a name in the original paper, we use GS-2013 for convenient description in this study), one locus was reported for virulence or avirulence phenotypes on the *Yr1, Yr7, Yr8, Yr27, YrExp2*, and *YrSP* wheat lines and two loci for wheat lines possessing *Yr2, Yr4, Yr6, Yr9, Yr28, Yr32, Yr43, Yr44, Yr76*, and *YrTr1* (Tian et al., 2017). By selfing a US isolate representing race PSTv-11, (Yuan et al., 2017) determined that the VPs on wheat lines of *Yr1, Yr6, Yr7, Yr8, Yr9, Yr25, Yr27, Yr28*, and *YrA* (*Yr73* and *Yr74*) were controlled by a single locus while those on lines with *Yr2, Yr17, Yr43, Yr44, Yr76*, and *YrExp2* controlled by two loci.

In general, the *Pst*-wheat interactions comply with the gene-for-gene hypothesis (Flor, 1971), which is supported by the one-locus controlled VP against some of the single *Yr*-gene near-isogenic lines. In contrast many of the virulence/avirulence phenotypes against other *Yr* “single-gene” lines were controlled by two loci in the pathogen in the present study and previous studies (Tian et al., 2016, 2017; Yuan et al., 2017) as discussed above. Multiple avirulence genes corresponding to one resistance gene have been reported in powdery mildew (Bourras et al., 2015, 2016). However, the genetics of multi-gene-controlled avirulence in *Pst* against a single *Yr* gene in wheat can be more complex because *Pst* is dikaryotic and interactions of genes in different nuclei could be different from those in a single nucleus. Further studies are needed to determine the genetic and molecular mechanisms of interactions between *Pst* virulence genes and wheat resistance genes.

Although it is possible that two virulence loci exist against a single resistance gene, many of the two-locus VPs identified in the present study and previous studies of *Pst* segregating populations can be explained by additional genes in the wheat *Yr*-gene lines used in these studies. There is no a near-isogenic line developed for *Yr2*. Because of lack of near-isogenic line for *Yr2*, this gene is not included in the core set differential, but in the supplementary set of differentials, represented by the wheat cultivar Kalyansona (Wan and Chen, 2014). In the present study and Tian et al. (2016), the number of loci for the virulence to *Yr2* was not determined, but the two loci for virulence to the *Yr2* were shown in the GS-2013 and PSTv-11 population (Tian et al., 2017; Yuan et al., 2017). Their results suggest that the *Yr2* wheat genotype may have an additional gene. The wheat cultivar Hybrid 46 used in this study to represent *Yr4* has at least one additional gene (*YrH46*) (Wan and Chen, 2014). Other *Yr*-gene lines, which detected segregation of virulence or avirulence, are in the Avocet S (AvS) background. *YrA* was determined to have two complementary genes (*Yr73* and *Yr74*) for resistance to some Australian *Pst* races (Dracatos et al., 2016). The two complementary genes detected by the *YrA* line in the present study support the two complementary resistance genes. Different from the *YrA* line (Avocet), AvS is a selection from Australian wheat cultivar Avocet for its susceptibility to some *Pst* races to which Avocet is resistant; and AvS was reported to have *Yr74* but without *Yr73* (Dracatos et al., 2016), which was supported by the virulence study with a segregating population developed from an isolate of PSTv-11 (Yuan et al., 2017). Many of the *Yr* gene lines that detected two virulence loci in the presented study and previous studies should have *Yr74* from AvS. Whether *Yr74* can be detected or not depends upon the homozygosity or heterozygosity of the *avr74* locus in the parental population. Because of this, we conclude that the interactions between *Pst* avirulence genes and wheat *Yr* genes are largely follow the one-gene for one-gene model.

### Intragenic and intergenic interactions of virulence genes

The present study showed that virulence or avirulence can be controlled by dominant or recessive genes. Although no a single dominant gene for avirulence was detected in the present study and the study of the population from a US isolate (Yuan et al., 2017), dominant single genes were detected for avirulence to *Yr6, Yr43*, and *YrSP* in the Pinglan 17-7 population (Tian et al., 2016) and to *Yr7, Yr8, YrSP*, and *YrExp2* in the GS-2013 population (Tian et al., 2017). Avirulence phenotypes controlled by two dominant genes were found on the *YrSP* line in the present study and on the *Yr4, Yr32*, and *YrTr1* lines in the GS-2013 population (Tian et al., 2017).

More dominant genes for virulence, rather than avirulence, were detected in the present study and the previous studies (Tian et al., 2016, 2017; Yuan et al., 2017). In the present study, all detected genes except those for avirulence to *YrSP*, were dominant for virulence or recessive for avirulence. Similarly, all genes were dominant for virulence in the population of US race PSTv-11 (Yuan et al., 2017). From the evolutionary standard point, dominant virulence genes provide the obligate parasite advantages for survival and growth on host plants. Without active gene products for virulence, the ability to overcome race specific resistance genes, *Pst* cannot infect and grow on host plants. Future studies are needed to clone dominant virulence genes and study their molecular interactions with corresponding resistance genes.

### Differences in inheritance of virulence phenotypes

The comparisons of virulence/avirulence inheritance of the CYR32 population in the present study with those in other populations (Table 4) allow us to identify consistently homozygous avirulence (i.e., those corresponding to *Yr5* and *Yr15* in both China and the U.S.) or virulence genes (i.e., virulence to *Yr25* in China). The consistent results indicated the usefulness of *Yr5* and *Yr15* in both countries and no value of *Yr25* in China. The findings of different genetic states for other VPs are also useful for predicting virulence changes and determining the usefulness of the corresponding resistance genes in breeding programs.

In the present study, the avirulence phenotype to *Yr7* was recessive, in consistent with the study of the population of US race PSTv-11 (Yuan et al., 2017), however, the avirulence phenotype to *Yr7* was found to be dominant in the GS-2013 population (Tian et al., 2017) (Table 4). Switches from recessive to dominant avirulence were observed for *Yr43* in the CYR32 population compared to the Pinglan 17-7 population (Tian et al., 2016); for *YrExp2* in the CYR32 population compared to the GS-2013 population (Tian et al., 2017); for *Yr8* in the PSTv-11 population (Yuan et al., 2017) compared with the GS-2013 population (Tian et al., 2017); and for *Yr6* in the PSTv-11 population compared with the Pinglan 17-7 population. When two loci were involved, switches of dominance to recessiveness were also observed. For example, a 1:15 ratio (two complementary and recessive genes for avirulence) for *Yr9* was observed in the CYR32 population whereas a 3:13 ratio (a dominant gene and a recessive gene in complementary) was observed in the GS-2013 population (Tian et al., 2017). Similarly, a 3:13 ratio was obtained in the CYR32 population for *Yr17* in this study while a 7:9 ratio (two independent recessive genes for virulence) was reported in the PSTv-11 population (Yuan et al., 2017). These differences in dominance and intergenic interactions indicate that the different *Pst* isolates may have different alleles at the virulence/avirulence loci and the different alleles control different phenotypes and interact differently with alleles at the other locus. Furthermore, virulence in one isolate was found to be controlled by a single gene, but in another isolate was controlled by two genes (Table 4). For example, the virulence to *Yr6* was controlled by two dominant genes (or the avirulence was controlled by two complementary recessive genes) in the CYR32 population in the present study and the GS-2013 population (Tian et al., 2017), but was controlled by a recessive gene in the Pinglan 17-7 population (Tian et al., 2016) and a dominant gene in the PSTv-11 population (Yuan et al., 2017). Thus, the genetic studies so far have identified a large number of virulence/avirulence loci; many with multiple alleles; and complex interactions between alleles at the same locus and different loci in different isolates and with host resistance genes. Further studies are needed to clone the individual alleles of virulence/avirulence and determine how they interact differently with their corresponding resistance genes. Such studies should generate information for understanding the pathogen-plant interactions and virulence-gene based markers for monitoring the pathogen populations and searching new genes for effective resistance to stripe rust.

### Linage of virulence/avirulence genes

In the present study, we constructed two linkages. Ten virulence/avirulence genes were mapped to linkage 1 with various genetic distances between two neighboring virulence loci. Avirulence to *YrSP* was segregated at a 9: 7 ratio, indicating the avirulence was controlled by two complementary dominant genes, *AvrSP1* and *AvrSP2*, which should be located in two different chromosomes. However, only one avirulent gene, namely *AvrSP1*, was mapped, but *AvrSP2* was not mapped due to the few markers in this study.

Linked virulence genes were also reported in previous studies (Tian et al., 2016, 2017; Yuan et al., 2017). Yuan et al. (2017) constructed a base genetic map for *Pst* including six chromosomes with 805 markers. Moreover, the parental isolate PSTv-11 was heterozygous for 19 virulence loci. The large number of markers and segregating virulence loci allowed the authors to establish a high resolution map and identified linkage of numerous virulence loci. In the present study, the small number of markers and relatively low heterozygosity of the parental isolate limited the construction of genetic linkage map. However, the linkage of virulence genes to *Yr6, Yr7, Yr9, Yr17, Yr27*, and *Yr28* identified in the present study is similar to the report of Yuan et al. (2017). They mapped these genes in a region of chromosome 6. This linkage may make cloning of these virulence genes relatively easy. More studies are needed to identify molecular markers for high-resolution mapping and genomically characterizing this chromosomal region.

Together with previous studies (Tian et al., 2016, 2017; Yuan et al., 2017), the present study demonstrates that a single race with heterozygous avirulence loci can generate through sexual reproduction a large number of different races, which may overcome genes in wheat cultivars for resistance to the single race. Thus, it is important to select effective resistance genes, such as *Yr5* and *Yr15* tested in the present study and many other *Yr* genes (Wang and Chen, 2017) based on the knowledge of homozygosity and heterozygosity of the corresponding avirulence genes in the pathogen populations. It is better to pyramid several effective *Yr* genes to develop high level and long-lasting resistant cultivars. It is even better to combine genes for partial but durable resistance with effective race-specific resistance genes (Wang and Chen, 2017). However, the determination of durability and race specificity of resistance genes relies on clearly defined races and populations of the pathogen (Chen, 2013). The results of the present study are useful for identifying effective and durable resistance genes to be used in developing wheat cultivars with adequate and durable resistance for sustainable control of stripe rust.

## Author contributions

ZK, JZ, LH, and LW: designed experiments; LW, SZ, and DZ: performed the experiments; LW and HZ: analyzed the data; LW, JZ, and XC: discussed and developed the study; LW, XC, ZK, and JZ: wrote the paper.

### Conflict of interest statement

The authors declare that the research was conducted in the absence of any commercial or financial relationships that could be construed as a potential conflict of interest.

## Supplementary material

Supplementary Figure 1 Genotypes of SSR marker scaffold 962-172974 in the parental isolate **(A)** and progeny isolates SA11 **(B)**, SA29 **(C)**, and SA75 **(D)**.

Supplementary Table 1 Wheat genotypes used in the study.

Supplementary Table 2 Virulence phenotypes (VP) of the parental race CYR32 and 127 progeny isolates of *Puccinia striiformis* f. sp. *tritici* on 25 wheat *Yr* gene lines.

Supplementary Table 3 Multi-locus genotypes (MLG) of the parental isolate CYR32 and progeny isolates determined based on 10 heterozygous SSR loci.
