# Supplementary figures and images for: Inheritance and Linkage of Virulence Genes in Chinese Predominant Race CYR32 of the Wheat Stripe Rust Pathogen Puccinia striiformis f. sp. tritici
Source: Front Plant Sci. 2018 Feb 8;9:120. doi: 10.3389/fpls.2018.00120 (PMC5809510; doi:10.3389/fpls.2018.00120)

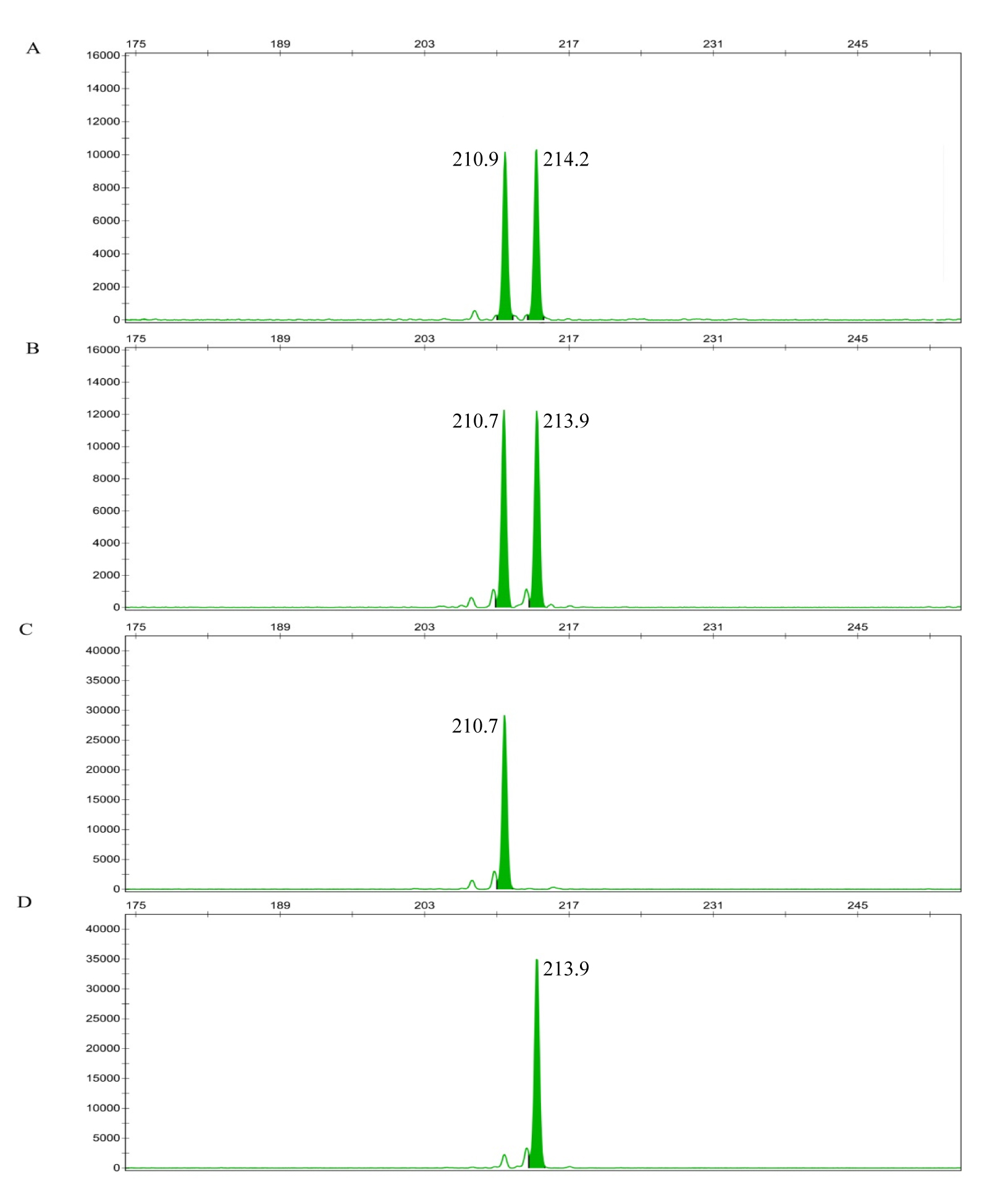

Supplement: Supplementary Figure 1 — Genotypes of SSR marker scaffold 962-172974 in the parental isolate (A) and progeny isolates SA11 (B), SA29 (C), and SA75 (D). [file Image1.JPEG]
