# Supplementary material for: Inheritance and Linkage of Virulence Genes in Chinese Predominant Race CYR32 of the Wheat Stripe Rust Pathogen Puccinia striiformis f. sp. tritici
Source: Front Plant Sci. 2018 Feb 8;9:120. doi: 10.3389/fpls.2018.00120 (PMC5809510; doi:10.3389/fpls.2018.00120)
Supplement: Supplementary Table 1 — Wheat genotypes used in the study. [file Table1.DOCX]

**Supplementary Table 1** | Wheat genotypes used in this study.

| No. | Wheat genotype | *Yr* gene | Growth habit | Differential | Reference |
| --- | --- | --- | --- | --- | --- |
| 1 | AvSYr1NIL | *Yr1* | Spring | US differential 1 | Wan and Chen, 2014 |
| 2 | AvSYr5NIL | *Yr5* | Spring | US differential 2 | Wan and Chen, 2014 |
| 3 | AvSYr6NIL | *Yr6* | Spring | US differential 3 | Wan and Chen, 2014 |
| 4 | AvSYr7NIL | *Yr7* | Spring | US differential 4 | Wan and Chen, 2014 |
| 5 | AvSYr8NIL | *Yr8* | Spring | US differential 5 | Wan and Chen, 2014 |
| 6 | AvSYr9NIL | *Yr9* | Spring | US differential 6 | Wan and Chen, 2014 |
| 7 | AvSYr10NIL | *Yr10* | Spring | US differential 7 | Wan and Chen, 2014 |
| 8 | AvSYr15NIL | *Yr15* | Spring | US differential 8 | Wan and Chen, 2014 |
| 9 | AvSYr17NIL | *Yr17* | Spring | US differential 9 | Wan and Chen, 2014 |
| 10 | AvSYr24NIL | *Yr24* | Spring | US differential 10 | Wan and Chen, 2014 |
| 11 | AvSYr27NIL | *Yr27* | Spring | US differential 11 | Wan and Chen, 2014 |
| 12 | AvSYr32NIL | *Yr32* | Spring | US differential 12 | Wan and Chen, 2014 |
| 13 | AvS/IDO377s (F3-41-1) | *Yr43* | Spring | US differential 13 | Wan and Chen, 2014 |
| 14 | AvS/Zak (1-1-35-line1 | *Yr44* | Spring | US differential 14 | Wan and Chen, 2014 |
| 15 | AvSYrSPNIL | *YrSP* | Spring | US differential 15 | Wan and Chen, 2014 |
| 16 | AvSYrTr1NIL | *YrTr1* | Spring | US differential 16 | Wan and Chen, 2014 |
| 17 | AvS/Exp 1/1-1 Line 74 | *YrExp2* | Spring | US differential 17 | Wan and Chen, 2014 |
| 18 | Tyee | *Yr76* (*YrTye*) | Winter | US differential 18 | Wan and Chen, 2014 |
| 19 | Kalyansona | *Yr2* | Spring | US Sup. differential 1 | Wan and Chen, 2014 |
| 20 | Vilmorin 23 | *Yr3* (*Yr4a*, *YrV23*) | Winter | US Sup. differential 2 | Wan and Chen, 2014 |
| 21 | Hybrid 46 | *Yr4b*, *YrH46* | Winter | US Sup. differential 3 | Wan and Chen, 2014 |
| 22 | Hugenoot | *Yr25* | Spring | US Sup. differential 4 | Wan and Chen, 2014 |
| 23 | AvSYr28NIL | *Yr28* | Spring | US Sup. differential 5 | Wan and Chen, 2014 |
| 24 | Avocet | *YrA* (*Yr73*, *Yr74*) | Spring | US Sup. differential 6 | Wan and Chen, 2014 |
| 25 | AvSYr26NIL | *Yr26* (=*Yr24*) | Spring | No | Wang and Chen, 2017 |
| 26 | Mingxian 169 | No | Winter | Susceptible check | Wan et al., 2004 |
