# Supplementary material for: Inheritance and Linkage of Virulence Genes in Chinese Predominant Race CYR32 of the Wheat Stripe Rust Pathogen Puccinia striiformis f. sp. tritici
Source: Front Plant Sci. 2018 Feb 8;9:120. doi: 10.3389/fpls.2018.00120 (PMC5809510; doi:10.3389/fpls.2018.00120)
Supplement: Supplementary Table 2 — Virulence phenotypes (VP) of the parental race CYR32 and 127 progeny isolates of Puccinia striiformis f. sp. tritici on 25 wheat Yr gene lines. [file Table2.DOCX]

**Supplementary Table 2** | Virulence phenotypes (VP) of the parental race CYR32 and 127 progeny isolates of *Puccinia striiformis* f. sp. *tritici* on 25 wheat *Yr* gene lines. ^a^

|  | Avirulence (A) and virulence (V) on *Yr* single-gene line | | | | | | | | | |  |
| --- | --- | --- | --- | --- | --- | --- | --- | --- | --- | --- | --- |
| Isolate | *Yr6* | *Yr7* | *Yr9* | *Yr17* | *Yr27* | *Yr28* | *Yr43* | *YrA* | *YrExp2* | *YrSP* | VP |
| CYR32 | V | V | V | V | V | V | V | V | V | A | VP 1 |
| SA 1 | V | V | V | V | V | V | V | V | V | V | VP 2 |
| SA 3 | V | V | V | V | V | V | V | V | V | A | VP 1 |
| SA 4 | V | V | V | V | V | V | V | V | V | A | VP 1 |
| SA 5 | V | V | V | V | V | V | V | V | V | A | VP 1 |
| SA 6 | V | A | A | A | A | A | A | V | A | A | VP 24 |
| SA 7 | V | V | V | V | V | V | V | V | V | V | VP 2 |
| SA 9 | V | V | V | V | V | V | V | V | V | A | VP 1 |
| SA 10 | V | V | V | V | V | V | V | V | V | V | VP 2 |
| SA 11 | V | A | V | V | V | V | V | V | V | A | VP 5 |
| SA 12 | V | V | V | V | V | A | V | V | V | A | VP 7 |
| SA 13 | V | V | V | V | V | V | V | V | V | V | VP 2 |
| SA 14 | V | V | V | V | V | V | V | V | V | V | VP 2 |
| SA 15 | V | V | V | V | V | V | V | V | V | A | VP 1 |
| SA 16 | V | V | V | V | V | V | V | V | A | A | VP 9 |
| SA 17 | V | V | V | V | V | V | V | V | V | A | VP 1 |
| SA 18 | A | A | A | A | A | A | A | A | A | A | VP 27 |
| SA 19 | V | A | V | A | A | A | A | A | A | A | VP 25 |
| SA 20 | V | V | V | V | V | V | A | V | V | A | VP 8 |
| SA 21 | V | V | V | V | V | A | V | V | V | A | VP 7 |
| SA 22 | V | V | V | V | V | A | V | V | V | A | VP 7 |
| SA 23 | V | A | A | A | A | A | A | V | A | A | VP 24 |
| SA 24 | V | V | V | V | V | V | V | V | V | V | VP 2 |
| SA 25 | V | V | V | V | V | V | V | V | V | V | VP 2 |
| SA 26 | V | V | V | V | V | V | V | V | V | V | VP 2 |
| SA 27 | V | V | V | V | V | V | V | V | V | V | VP 2 |
| SA 29 | A | A | A | A | A | A | A | A | A | A | VP 27 |
| SA 31 | V | A | V | V | V | V | A | V | V | A | VP 11 |
| SA 32 | V | A | V | V | V | V | A | V | A | A | VP 17 |
| SA 33 | V | A | V | V | V | A | A | V | A | A | VP 18 |
| SA 34 | V | A | V | V | V | V | A | V | A | A | VP 17 |
| SA 35 | V | V | V | V | V | V | A | V | A | A | VP 16 |
| SA 36 | V | V | V | V | V | V | A | V | A | A | VP 16 |
| SA 37 | A | A | A | A | A | A | A | A | A | A | VP 27 |
| SA 38 | V | A | V | V | V | V | A | V | A | A | VP 17 |
| SA 39 | V | A | V | A | V | A | A | A | A | A | VP 22 |
| SA 40 | V | V | V | V | V | V | A | V | A | A | VP 16 |
| SA 42 | V | V | V | V | V | V | V | V | V | A | VP 1 |
| SA 43 | V | V | V | V | V | V | V | V | V | A | VP 1 |
| SA 44 | V | V | V | V | V | V | V | V | V | V | VP 2 |
| SA 45 | V | V | V | V | V | V | V | V | V | V | VP 2 |
| SA 46 | V | V | V | V | V | V | V | V | V | A | VP 1 |
| SA 47 | V | V | V | V | V | V | V | V | V | A | VP 1 |
| SA 48 | V | V | V | V | V | V | V | V | V | A | VP 1 |
| SA 49 | V | V | V | V | V | V | V | V | A | A | VP 9 |
| SA 50 | V | V | V | V | V | V | V | V | V | A | VP 1 |
| SA 51 | V | V | V | V | V | V | V | V | V | A | VP 1 |
| SA 52 | V | V | V | V | V | V | V | V | V | A | VP 1 |
| SA 53 | V | V | V | V | V | V | V | V | V | A | VP 1 |
| SA 57 | V | V | V | V | V | V | V | V | V | A | VP 1 |
| SA 58 | V | V | V | V | V | V | V | V | V | A | VP 1 |
| SA 59 | V | V | V | V | V | V | V | V | V | V | VP 2 |
| SA 61 | V | V | V | V | V | V | V | V | V | A | VP 1 |
| SA 62 | V | V | V | V | V | V | V | V | V | A | VP 1 |
| SA 63 | V | V | V | V | V | V | V | V | V | V | VP 2 |
| SA 64 | V | V | V | V | V | V | V | V | V | A | VP 1 |
| SA 65 | V | V | V | V | V | V | V | V | V | V | VP 2 |
| SA 66 | V | V | V | V | V | V | V | V | V | V | VP 2 |
| SA 67 | V | V | V | V | V | V | V | V | V | V | VP 2 |
| SA 68 | V | V | V | A | V | V | V | V | V | A | VP 6 |
| SA 69 | V | V | V | V | A | A | V | V | A | V | VP 14 |
| SA 70 | V | V | V | V | V | A | A | V | A | V | VP 15 |
| SA 71 | V | V | V | V | V | V | A | V | V | V | VP 3 |
| SA 72 | V | V | V | V | A | A | A | V | A | A | VP 19 |
| SA 73 | V | A | A | A | A | A | A | V | A | V | VP 21 |
| SA 75 | V | V | V | V | V | V | A | V | V | V | VP 3 |
| SA 76 | V | A | V | V | V | V | A | V | A | V | VP 10 |
| SA 77 | V | V | V | V | V | V | V | V | V | V | VP 2 |
| SA 78 | V | A | V | V | V | V | V | V | A | V | VP 4 |
| SA 79 | V | V | V | V | V | V | V | V | V | V | VP 2 |
| SA 80 | V | V | V | V | V | V | V | V | V | V | VP 2 |
| SA 81 | V | V | V | V | V | V | V | V | V | V | VP 2 |
| SA 82 | V | V | V | V | V | V | V | V | V | V | VP 2 |
| SA 83 | V | V | V | V | V | V | V | V | V | V | VP 2 |
| SA 84 | V | V | A | A | A | A | A | A | A | V | VP 23 |
| SA 85 | V | V | V | V | V | V | V | V | V | V | VP 2 |
| SA 86 | V | V | V | V | V | V | V | V | V | V | VP 2 |
| SA 87 | V | V | V | V | V | V | V | V | V | V | VP 2 |
| SA 88 | V | V | V | V | V | V | V | V | V | V | VP 2 |
| SA 89 | V | V | V | V | V | V | V | V | V | V | VP 2 |
| SA 90 | V | V | V | V | V | V | V | V | V | V | VP 2 |
| SA 91 | V | V | V | V | V | V | V | V | V | V | VP 2 |
| SA 92 | V | V | V | V | V | V | V | V | V | A | VP 1 |
| SA 93 | V | V | V | V | V | V | V | V | V | A | VP 1 |
| SA 94 | V | V | V | V | V | V | V | V | V | A | VP 1 |
| SA 95 | V | V | V | V | V | A | V | V | V | A | VP 7 |
| SA 96 | V | V | V | V | V | V | V | V | V | V | VP 2 |
| SA 97 | V | A | V | V | V | A | A | V | A | A | VP 18 |
| SA 99 | A | A | A | A | A | A | V | A | A | A | VP 26 |
| SA 100 | V | V | V | V | V | V | V | V | V | V | VP 2 |
| SA 101 | V | V | V | V | V | V | V | V | V | V | VP 2 |
| SA 102 | V | V | V | V | V | V | V | V | V | V | VP 2 |
| SA 103 | V | V | V | V | V | V | V | V | V | V | VP 2 |
| SA 104 | V | V | V | V | V | V | V | V | V | V | VP 2 |
| SA 106 | V | V | V | V | V | V | V | V | V | V | VP 2 |
| SA 107 | A | A | A | A | A | A | A | A | A | A | VP 27 |
| SA 108 | V | V | V | V | V | V | V | V | V | V | VP 2 |
| SA 109 | V | V | V | V | V | V | A | V | V | A | VP 8 |
| SA 110 | V | V | V | V | V | V | V | V | V | V | VP 2 |
| SA 112 | V | A | V | V | V | V | A | V | V | A | VP 11 |
| SA 114 | A | A | A | A | A | A | A | A | A | A | VP 27 |
| SA 115 | V | A | V | V | V | V | A | V | V | A | VP 11 |
| SA 116 | V | V | V | V | V | V | V | V | V | V | VP 2 |
| SA 117 | V | A | V | V | V | V | A | V | A | V | VP 10 |
| SA 119 | V | A | V | V | V | V | A | V | V | A | VP 11 |
| SA 120 | V | V | V | V | V | V | A | V | V | V | VP 3 |
| SA 121 | V | V | V | V | V | V | V | V | V | A | VP 1 |
| SA 122 | V | V | V | V | V | A | V | V | V | A | VP 7 |
| SA 123 | A | A | A | A | A | A | A | A | A | A | VP 27 |
| SA 125 | V | V | V | V | V | V | V | V | V | A | VP 1 |
| SA 127 | V | V | V | V | V | A | V | V | V | A | VP 7 |
| SA 128 | V | A | V | V | V | V | V | V | A | A | VP 12 |
| SA 131 | V | V | V | V | V | V | V | V | V | V | VP 3 |
| SA 132 | V | V | V | V | V | V | V | V | V | A | VP 1 |
| SA 133 | V | V | V | V | V | V | V | V | V | A | VP 1 |
| SA 134 | V | V | V | V | V | V | V | V | V | A | VP 1 |
| SA 135 | V | A | V | A | V | A | A | V | A | A | VP 20 |
| SA 136 | V | V | V | V | V | V | V | V | V | A | VP 1 |
| SA 137 | V | V | V | V | V | V | V | V | V | V | VP 3 |
| SA 138 | V | V | V | V | V | V | A | V | A | A | VP 16 |
| SA 139 | V | V | V | V | V | V | V | V | V | A | VP 1 |
| SA 141 | V | V | V | V | V | V | V | V | V | A | VP 1 |
| SA 142 | V | V | V | V | V | V | V | V | A | A | VP 9 |
| SA 144 | A | A | A | A | A | A | A | A | A | A | VP 27 |
| SA 146 | V | V | V | V | V | V | V | V | V | V | VP 3 |
| SA 147 | V | V | V | V | V | V | V | V | V | A | VP 1 |
| SA 148 | V | V | A | V | V | V | V | V | A | A | VP 13 |
| SA 149 | V | V | V | V | V | V | V | V | V | A | VP 1 |

^a^ The parental isolate CYR32 and the progeny isolates were tested on 25 wheat *Yr* gene lines. CYR32 and all progeny isolates were avirulent (infection types 0-6) to *Yr5*, *Yr8*, *Yr10*, *Yr15*, *Yr24*, *Yr26*, *Yr32* and *YrTr1*, and virulent (infection types 7-9) to *Yr1*, *Yr2*, *Yr3*, *Yr4*, *Yr25*, *Yr44*, and *Yr76* (*YrTye*). SA = single-aeciospore isolate. VP = virulence phenotype.
