# Supplementary material for: Inheritance and Linkage of Virulence Genes in Chinese Predominant Race CYR32 of the Wheat Stripe Rust Pathogen Puccinia striiformis f. sp. tritici
Source: Front Plant Sci. 2018 Feb 8;9:120. doi: 10.3389/fpls.2018.00120 (PMC5809510; doi:10.3389/fpls.2018.00120)
Supplement: Supplementary Table 3 — Multi-locus genotypes (MLG) of the parental isolate CYR32 and progeny isolates determined based on 10 heterozygous SSR loci. [file Table3.DOCX]

**Supplementary Table 3** | Multi-locus genotypes (MLG) of the parental isolate CYR32 and progeny isolates determined based on 10 heterozygous SSR loci.

|  | Amplified fragment (base pairs) of SSR markers ^a^ | | | | | | | | | | | | | | | | | | | |  |  |
| --- | --- | --- | --- | --- | --- | --- | --- | --- | --- | --- | --- | --- | --- | --- | --- | --- | --- | --- | --- | --- | --- | --- |
| Isolate | M1 | | M2 | | M3 | | M4 | | M5 | | M6 | | M7 | | M8 | | M9 | | M10 | | MLG | No. of segregated markers |
|  |  |  |  |  |  |  |  |  |  |  |  |  |  |  |  |  |  |  |  |  |  |  |
|  | a1 | a2 | a1 | a2 | a1 | a2 | a1 | a2 | a1 | a2 | a1 | a2 | a1 | a2 | a1 | a2 | a1 | a2 | a1 | a2 |  |  |
| CYR32 | 315 | 318 | 286 | 298 | 237 | 253 | 274 | 288 | 211 | 214 | 224 | 233 | 231 | 253 | 357 | 365 | 339 | 349 | 308 | 323 | MLG 1 | 10 |
| SA 1 | 315 | 315 | 286 | 298 | 237 | 253 | 274 | 274 | 214 | 214 | 233 | 233 | 231 | 253 | 365 | 365 | 339 | 349 | 308 | 308 | MLG 51 | 4 |
| SA 3 | 315 | 318 | 286 | 298 | 253 | 253 | 288 | 288 | 211 | 214 | 233 | 233 | 231 | 253 | 357 | 357 | 349 | 349 | 323 | 323 | MLG 40 | 4 |
| SA 4 | 315 | 318 | 286 | 298 | 253 | 253 | 288 | 288 | 211 | 214 | 233 | 233 | 231 | 253 | 357 | 357 | 349 | 349 | 323 | 323 | MLG 40 | 4 |
| SA 5 | 315 | 318 | 286 | 286 | 253 | 253 | 274 | 288 | 211 | 211 | 224 | 224 | 231 | 253 | 357 | 357 | 349 | 349 | 323 | 323 | MLG 58 | 3 |
| SA 6 | 315 | 318 | 286 | 298 | 237 | 237 | 274 | 274 | 214 | 214 | 224 | 224 | 231 | 253 | 357 | 365 | 349 | 349 | 323 | 323 | MLG 43 | 4 |
| SA 7 | 318 | 318 | 286 | 286 | 253 | 253 | 274 | 288 | 211 | 211 | 224 | 233 | 231 | 253 | 357 | 365 | 339 | 349 | 308 | 323 | MLG 19 | 6 |
| SA 9 | 315 | 318 | 286 | 286 | 237 | 253 | 288 | 288 | 214 | 214 | 224 | 224 | 231 | 231 | 365 | 365 | 339 | 339 | 308 | 308 | MLG 64 | 2 |
| SA 10 | 315 | 318 | 286 | 298 | 237 | 253 | 288 | 288 | 211 | 214 | 224 | 224 | 231 | 253 | 357 | 357 | 339 | 339 | 308 | 308 | MLG 36 | 5 |
| SA 11 | 315 | 318 | 286 | 298 | 237 | 253 | 274 | 288 | 211 | 214 | 224 | 233 | 231 | 253 | 357 | 365 | 339 | 349 | 308 | 323 | MLG 1 | 10 |
| SA 12 | 318 | 318 | 286 | 298 | 237 | 253 | 288 | 288 | 211 | 214 | 224 | 233 | 253 | 253 | 357 | 365 | 339 | 339 | 308 | 308 | MLG 26 | 5 |
| SA 13 | 315 | 318 | 286 | 298 | 253 | 253 | 288 | 288 | 211 | 214 | 233 | 233 | 231 | 253 | 357 | 357 | 349 | 349 | 323 | 323 | MLG 40 | 4 |
| SA 14 | 318 | 318 | 286 | 298 | 237 | 253 | 274 | 274 | 211 | 214 | 224 | 233 | 231 | 231 | 365 | 365 | 339 | 339 | 308 | 308 | MLG 47 | 4 |
| SA 15 | 315 | 318 | 286 | 298 | 253 | 253 | 288 | 288 | 211 | 214 | 233 | 233 | 231 | 253 | 357 | 357 | 349 | 349 | 323 | 323 | MLG 40 | 4 |
| SA 16 | 318 | 318 | 286 | 298 | 237 | 237 | 288 | 288 | 214 | 214 | 224 | 233 | 231 | 231 | 357 | 357 | 339 | 339 | 308 | 323 | MLG 62 | 3 |
| SA 17 | 318 | 318 | 286 | 298 | 237 | 253 | 274 | 274 | 211 | 214 | 224 | 233 | 231 | 231 | 365 | 365 | 339 | 339 | 308 | 308 | MLG 47 | 4 |
| SA 18 | 318 | 318 | 286 | 298 | 237 | 253 | 274 | 288 | 211 | 214 | 224 | 224 | 253 | 253 | 365 | 365 | 339 | 349 | 308 | 323 | MLG 17 | 6 |
| SA 19 | 315 | 315 | 286 | 298 | 237 | 253 | 274 | 274 | 214 | 214 | 233 | 233 | 231 | 253 | 365 | 365 | 339 | 349 | 308 | 308 | MLG 51 | 4 |
| SA 20 | 315 | 318 | 286 | 298 | 253 | 253 | 274 | 288 | 211 | 214 | 224 | 233 | 253 | 253 | 357 | 357 | 349 | 349 | 323 | 323 | MLG 38 | 5 |
| SA 21 | 315 | 315 | 286 | 298 | 237 | 253 | 274 | 274 | 214 | 214 | 224 | 224 | 231 | 253 | 365 | 365 | 339 | 349 | 308 | 308 | MLG 50 | 4 |
| SA 22 | 315 | 318 | 286 | 298 | 253 | 253 | 274 | 274 | 211 | 214 | 224 | 233 | 253 | 253 | 357 | 357 | 349 | 349 | 323 | 323 | MLG 42 | 4 |
| SA 23 | 315 | 315 | 286 | 298 | 253 | 253 | 274 | 288 | 211 | 214 | 233 | 233 | 231 | 253 | 357 | 365 | 339 | 349 | 308 | 323 | MLG 8 | 7 |
| SA 24 | 315 | 318 | 286 | 298 | 253 | 253 | 288 | 288 | 211 | 214 | 224 | 233 | 231 | 253 | 357 | 357 | 349 | 349 | 323 | 323 | MLG 37 | 5 |
| SA 25 | 318 | 318 | 286 | 286 | 237 | 253 | 274 | 274 | 211 | 214 | 224 | 233 | 231 | 253 | 357 | 365 | 339 | 349 | 308 | 323 | MLG 9 | 7 |
| SA 26 | 315 | 318 | 286 | 298 | 237 | 253 | 274 | 288 | 211 | 214 | 224 | 224 | 231 | 253 | 357 | 365 | 339 | 339 | 308 | 308 | MLG 11 | 7 |
| SA 27 | 315 | 315 | 286 | 298 | 237 | 253 | 274 | 274 | 214 | 214 | 233 | 233 | 231 | 253 | 365 | 365 | 339 | 349 | 308 | 308 | MLG 51 | 4 |
| SA 29 | 315 | 318 | 286 | 286 | 237 | 237 | 274 | 274 | 211 | 211 | 224 | 233 | 231 | 231 | 357 | 365 | 339 | 349 | 308 | 323 | MLG 30 | 5 |
| SA 31 | 315 | 318 | 286 | 298 | 253 | 253 | 288 | 288 | 211 | 214 | 233 | 233 | 231 | 253 | 357 | 357 | 349 | 349 | 323 | 323 | MLG 40 | 4 |
| SA 32 | 315 | 318 | 286 | 298 | 237 | 253 | 274 | 288 | 211 | 214 | 224 | 233 | 231 | 253 | 357 | 365 | 339 | 339 | 308 | 308 | MLG 4 | 8 |
| SA 33 | 315 | 318 | 286 | 298 | 237 | 237 | 274 | 288 | 211 | 214 | 224 | 224 | 231 | 231 | 365 | 365 | 339 | 349 | 308 | 323 | MLG 14 | 6 |
| SA 34 | 318 | 318 | 286 | 298 | 237 | 253 | 274 | 288 | 211 | 214 | 224 | 233 | 231 | 231 | 365 | 365 | 339 | 339 | 308 | 308 | MLG 28 | 5 |
| SA 35 | 315 | 318 | 286 | 286 | 253 | 253 | 274 | 288 | 211 | 214 | 224 | 233 | 231 | 231 | 357 | 357 | 349 | 349 | 323 | 323 | MLG 45 | 4 |
| SA 36 | 318 | 318 | 286 | 286 | 253 | 253 | 274 | 288 | 211 | 214 | 224 | 233 | 231 | 253 | 357 | 365 | 339 | 349 | 308 | 323 | MLG 6 | 7 |
| SA 37 | 315 | 315 | 286 | 298 | 237 | 253 | 274 | 274 | 214 | 214 | 233 | 233 | 231 | 253 | 365 | 365 | 339 | 349 | 308 | 308 | MLG 51 | 4 |
| SA 38 | 315 | 315 | 286 | 298 | 237 | 253 | 274 | 274 | 214 | 214 | 224 | 233 | 231 | 253 | 365 | 365 | 339 | 349 | 308 | 308 | MLG 22 | 5 |
| SA 39 | 315 | 318 | 286 | 298 | 253 | 253 | 288 | 288 | 211 | 214 | 224 | 233 | 231 | 253 | 357 | 357 | 349 | 349 | 323 | 323 | MLG 37 | 5 |
| SA 40 | 318 | 318 | 298 | 298 | 237 | 253 | 274 | 288 | 211 | 211 | 233 | 233 | 231 | 253 | 357 | 365 | 339 | 349 | 308 | 323 | MLG 20 | 6 |
| SA 42 | 315 | 318 | 286 | 298 | 253 | 253 | 288 | 288 | 211 | 214 | 224 | 233 | 231 | 253 | 357 | 357 | 349 | 349 | 323 | 323 | MLG 37 | 5 |
| SA 43 | 315 | 315 | 298 | 298 | 253 | 253 | 288 | 288 | 211 | 214 | 233 | 233 | 253 | 253 | 357 | 365 | 339 | 349 | 308 | 323 | MLG 54 | 4 |
| SA 44 | 315 | 318 | 286 | 298 | 253 | 253 | 288 | 288 | 211 | 214 | 233 | 233 | 231 | 253 | 357 | 357 | 349 | 349 | 323 | 323 | MLG 40 | 4 |
| SA 45 | 315 | 318 | 286 | 298 | 253 | 253 | 288 | 288 | 211 | 214 | 224 | 233 | 231 | 253 | 357 | 357 | 349 | 349 | 323 | 323 | MLG 37 | 5 |
| SA 46 | 315 | 318 | 286 | 286 | 253 | 253 | 274 | 288 | 211 | 211 | 224 | 224 | 231 | 253 | 357 | 357 | 349 | 349 | 323 | 323 | MLG 58 | 3 |
| SA 47 | 315 | 318 | 286 | 286 | 253 | 253 | 274 | 288 | 211 | 211 | 224 | 224 | 231 | 253 | 357 | 357 | 349 | 349 | 323 | 323 | MLG 58 | 3 |
| SA 48 | 318 | 318 | 286 | 298 | 237 | 253 | 274 | 288 | 211 | 214 | 224 | 224 | 253 | 253 | 365 | 365 | 339 | 349 | 323 | 323 | MLG 29 | 5 |
| SA 49 | 315 | 318 | 286 | 298 | 253 | 253 | 288 | 288 | 211 | 214 | 224 | 224 | 231 | 253 | 357 | 357 | 349 | 349 | 323 | 323 | MLG 39 | 4 |
| SA 50 | 315 | 318 | 286 | 286 | 253 | 253 | 274 | 288 | 211 | 211 | 224 | 233 | 231 | 253 | 357 | 357 | 349 | 349 | 323 | 323 | MLG 46 | 4 |
| SA 51 | 315 | 318 | 286 | 286 | 253 | 253 | 274 | 288 | 211 | 211 | 224 | 233 | 231 | 253 | 357 | 357 | 349 | 349 | 323 | 323 | MLG 46 | 4 |
| SA 52 | 315 | 315 | 286 | 298 | 237 | 253 | 274 | 274 | 214 | 214 | 233 | 233 | 231 | 253 | 365 | 365 | 339 | 349 | 308 | 308 | MLG 51 | 4 |
| SA 53 | 315 | 318 | 286 | 286 | 253 | 253 | 274 | 288 | 211 | 211 | 224 | 224 | 231 | 253 | 357 | 357 | 349 | 349 | 323 | 323 | MLG 58 | 3 |
| SA 57 | 315 | 318 | 286 | 286 | 253 | 253 | 274 | 288 | 211 | 211 | 224 | 224 | 231 | 253 | 357 | 357 | 349 | 349 | 323 | 323 | MLG 58 | 3 |
| SA 58 | 315 | 318 | 286 | 286 | 253 | 253 | 274 | 288 | 211 | 211 | 233 | 233 | 231 | 253 | 357 | 357 | 349 | 349 | 323 | 323 | MLG 59 | 3 |
| SA 59 | 315 | 318 | 286 | 286 | 253 | 253 | 274 | 288 | 211 | 211 | 224 | 233 | 231 | 253 | 357 | 357 | 349 | 349 | 323 | 323 | MLG 46 | 4 |
| SA 61 | 315 | 318 | 286 | 286 | 253 | 253 | 288 | 288 | 211 | 211 | 224 | 233 | 231 | 253 | 357 | 357 | 349 | 349 | 323 | 323 | MLG 56 | 3 |
| SA 62 | 315 | 318 | 286 | 286 | 253 | 253 | 274 | 288 | 211 | 211 | 224 | 224 | 231 | 253 | 357 | 357 | 349 | 349 | 323 | 323 | MLG 58 | 3 |
| SA 63 | NB | NB | 286 | 286 | 253 | 253 | NB | NB | 211 | 214 | 224 | 224 | 253 | 253 | NB | NB | 349 | 349 | NB | NB | MLG 65 | 1 |
| SA 64 | 315 | 318 | 286 | 286 | 253 | 253 | 274 | 288 | 211 | 211 | 224 | 224 | 231 | 253 | 357 | 357 | 349 | 349 | 323 | 323 | MLG 58 | 3 |
| SA 65 | 315 | 318 | 286 | 286 | 253 | 253 | 274 | 288 | 211 | 211 | 224 | 224 | 231 | 253 | 357 | 357 | 349 | 349 | 323 | 323 | MLG 58 | 3 |
| SA 66 | 315 | 318 | 286 | 286 | 253 | 253 | 274 | 288 | 211 | 211 | 224 | 233 | 231 | 253 | 357 | 357 | 349 | 349 | 323 | 323 | MLG 46 | 4 |
| SA 67 | 315 | 318 | 286 | 286 | 253 | 253 | 274 | 288 | 211 | 211 | 224 | 224 | 231 | 253 | 357 | 357 | 349 | 349 | 323 | 323 | MLG 58 | 3 |
| SA 68 | 315 | 318 | 286 | 286 | 253 | 253 | 274 | 288 | 211 | 211 | 224 | 233 | 231 | 253 | 357 | 357 | 349 | 349 | 323 | 323 | MLG 46 | 4 |
| SA 69 | 315 | 318 | 286 | 286 | 253 | 253 | 274 | 288 | 211 | 211 | 224 | 224 | 231 | 253 | 357 | 357 | 349 | 349 | 323 | 323 | MLG 58 | 3 |
| SA 70 | 315 | 318 | 286 | 286 | 253 | 253 | 274 | 288 | 211 | 211 | 224 | 224 | 231 | 253 | 357 | 357 | 349 | 349 | 323 | 323 | MLG 58 | 3 |
| SA 71 | 315 | 318 | 286 | 298 | 253 | 253 | 288 | 288 | 211 | 214 | 233 | 233 | 231 | 253 | 357 | 357 | 349 | 349 | 323 | 323 | MLG 40 | 4 |
| SA 72 | 318 | 318 | 286 | 298 | 237 | 253 | 274 | 288 | 211 | 214 | 224 | 233 | 253 | 253 | 365 | 365 | 339 | 349 | 323 | 323 | MLG 16 | 6 |
| SA 73 | 318 | 318 | 286 | 298 | 237 | 253 | 274 | 288 | 211 | 214 | 224 | 224 | 253 | 253 | 365 | 365 | 339 | 349 | 323 | 323 | MLG 29 | 5 |
| SA 75 | 315 | 318 | 286 | 298 | 237 | 237 | 274 | 274 | 214 | 214 | 224 | 224 | 231 | 253 | 357 | 365 | 349 | 349 | 323 | 323 | MLG 43 | 4 |
| SA 76 | 318 | 318 | 286 | 298 | 237 | 253 | 274 | 288 | 211 | 214 | 224 | 233 | 253 | 253 | 365 | 365 | 339 | 349 | 323 | 323 | MLG 16 | 6 |
| SA 77 | 318 | 318 | 286 | 286 | 253 | 253 | 274 | 288 | 211 | 211 | 224 | 233 | 231 | 253 | 357 | 365 | 339 | 349 | 308 | 323 | MLG 19 | 6 |
| SA 78 | 318 | 318 | 286 | 286 | 253 | 253 | 274 | 288 | 211 | 211 | 224 | 224 | 231 | 253 | 357 | 365 | 339 | 349 | 308 | 323 | MLG 25 | 5 |
| SA 79 | 318 | 318 | 286 | 286 | 253 | 253 | 274 | 288 | 211 | 211 | 224 | 224 | 231 | 253 | 357 | 365 | 339 | 349 | 308 | 323 | MLG 25 | 5 |
| SA 80 | 318 | 318 | 286 | 286 | 253 | 253 | 274 | 288 | 211 | 211 | 224 | 233 | 231 | 253 | 357 | 365 | 339 | 349 | 308 | 323 | MLG 19 | 6 |
| SA 81 | 318 | 318 | 286 | 286 | 253 | 253 | 274 | 288 | 211 | 211 | 224 | 233 | 231 | 253 | 357 | 365 | 339 | 349 | 308 | 323 | MLG 19 | 6 |
| SA 82 | 318 | 318 | 286 | 286 | 253 | 253 | 274 | 288 | 211 | 211 | 224 | 233 | 231 | 253 | 357 | 365 | 339 | 349 | 308 | 323 | MLG 19 | 6 |
| SA 83 | 318 | 318 | 286 | 286 | 253 | 253 | 274 | 288 | 211 | 211 | 224 | 233 | 231 | 253 | 357 | 365 | 339 | 349 | 308 | 323 | MLG 19 | 6 |
| SA 84 | 318 | 318 | 286 | 286 | 253 | 253 | 274 | 288 | 211 | 211 | 224 | 233 | 231 | 253 | 357 | 365 | 339 | 349 | 308 | 323 | MLG 19 | 6 |
| SA 85 | 318 | 318 | 286 | 286 | 253 | 253 | 274 | 288 | 211 | 211 | 224 | 233 | 231 | 253 | 357 | 365 | 339 | 349 | 308 | 323 | MLG 19 | 6 |
| SA 86 | 318 | 318 | 286 | 286 | 253 | 253 | 274 | 288 | 211 | 211 | 224 | 233 | 231 | 253 | 357 | 365 | 339 | 349 | 308 | 323 | MLG 19 | 6 |
| SA 87 | 318 | 318 | 286 | 286 | 253 | 253 | 274 | 288 | 211 | 211 | 224 | 233 | 231 | 253 | 357 | 365 | 339 | 349 | 308 | 323 | MLG 19 | 6 |
| SA 88 | 318 | 318 | 286 | 286 | 253 | 253 | 274 | 288 | 211 | 211 | 224 | 233 | 231 | 253 | 357 | 365 | 339 | 349 | 308 | 323 | MLG 19 | 6 |
| SA 89 | 318 | 318 | 286 | 286 | 253 | 253 | 274 | 288 | 211 | 211 | 224 | 224 | 231 | 253 | 357 | 365 | 339 | 349 | 308 | 323 | MLG 25 | 5 |
| SA 90 | 318 | 318 | 286 | 286 | 253 | 253 | 274 | 288 | 211 | 211 | 224 | 233 | 231 | 253 | 357 | 365 | 339 | 349 | 308 | 323 | MLG 19 | 6 |
| SA 91 | 318 | 318 | 286 | 286 | 253 | 253 | 274 | 288 | 211 | 211 | 224 | 233 | 231 | 253 | 357 | 365 | 339 | 349 | 308 | 323 | MLG 19 | 6 |
| SA 92 | 318 | 318 | 286 | 286 | 253 | 253 | 274 | 288 | 211 | 211 | 224 | 233 | 231 | 253 | 357 | 365 | 339 | 349 | 308 | 323 | MLG 19 | 6 |
| SA 93 | 315 | 318 | 298 | 298 | 237 | 253 | 274 | 274 | 214 | 214 | 224 | 224 | 231 | 253 | 357 | 365 | 339 | 349 | 323 | 323 | MLG 32 | 5 |
| SA 94 | 315 | 315 | 298 | 298 | 237 | 253 | 274 | 274 | 214 | 214 | NB | NB | 231 | 253 | 357 | 365 | 339 | 349 | 323 | 323 | MLG 53 | 4 |
| SA 95 | 315 | 318 | 286 | 298 | 253 | 253 | 288 | 288 | 211 | 214 | 233 | 233 | 231 | 253 | 357 | 357 | 349 | 349 | 323 | 323 | MLG 40 | 4 |
| SA 96 | 315 | 315 | 298 | 298 | 237 | 253 | 274 | 288 | 214 | 214 | 224 | 233 | 231 | 231 | 357 | 357 | 339 | 349 | 308 | 323 | MLG 21 | 5 |
| SA 97 | 318 | 318 | 286 | 298 | 237 | 253 | 274 | 288 | 211 | 214 | 224 | 224 | 253 | 253 | 365 | 365 | 339 | 349 | 323 | 323 | MLG 29 | 5 |
| SA 99 | 315 | 318 | 286 | 298 | 237 | 253 | 288 | 288 | 214 | 214 | 224 | 233 | 231 | 231 | 365 | 365 | 339 | 339 | 308 | 308 | MLG 41 | 4 |
| SA 100 | 318 | 318 | 286 | 298 | 237 | 253 | 274 | 288 | 211 | 214 | 224 | 233 | 253 | 253 | 365 | 365 | 339 | 349 | 323 | 323 | MLG 16 | 6 |
| SA 101 | 318 | 318 | 286 | 298 | 237 | 253 | 274 | 288 | 211 | 214 | 224 | 224 | 253 | 253 | 365 | 365 | 339 | 349 | 323 | 323 | MLG 29 | 5 |
| SA 102 | 318 | 318 | 286 | 298 | 237 | 253 | 274 | 288 | 211 | 214 | 224 | 224 | 253 | 253 | 365 | 365 | 339 | 349 | 323 | 323 | MLG 29 | 5 |
| SA 103 | 318 | 318 | 286 | 298 | 237 | 253 | 274 | 288 | 211 | 214 | 224 | 224 | 253 | 253 | 365 | 365 | 339 | 349 | 323 | 323 | MLG 29 | 5 |
| SA 104 | 315 | 315 | 286 | 298 | 237 | 253 | 274 | 288 | 214 | 214 | 224 | 224 | 253 | 253 | 357 | 365 | 339 | 349 | 308 | 323 | MLG 18 | 6 |
| SA 106 | 315 | 315 | 286 | 286 | 237 | 253 | 288 | 288 | 214 | 214 | 233 | 233 | 231 | 253 | 357 | 357 | 339 | 349 | 308 | 323 | MLG 52 | 4 |
| SA 107 | 315 | 318 | 298 | 298 | 237 | 253 | 288 | 288 | 211 | 211 | 224 | 224 | 231 | 253 | 357 | 357 | 339 | 339 | 308 | 308 | MLG 55 | 3 |
| SA 108 | 315 | 318 | 286 | 298 | 237 | 253 | 288 | 288 | 211 | 214 | 224 | 224 | 231 | 253 | 357 | 357 | 339 | 339 | 308 | 308 | MLG 36 | 5 |
| SA 109 | 318 | 318 | NB | NB | 237 | 253 | 274 | 288 | 211 | 211 | 224 | 224 | 231 | 253 | NB | NB | 339 | 339 | NB | NB | MLG 60 | 3 |
| SA 110 | 315 | 318 | 286 | 298 | 253 | 253 | 288 | 288 | 211 | 214 | 233 | 233 | 231 | 253 | 357 | 357 | 349 | 349 | 323 | 323 | MLG 40 | 4 |
| SA 112 | 315 | 318 | 286 | 298 | 237 | 253 | 288 | 288 | 211 | 214 | 224 | 224 | 231 | 253 | 357 | 357 | 339 | 339 | 308 | 308 | MLG 36 | 5 |
| SA 114 | 318 | 318 | 286 | 286 | 253 | 253 | 274 | 288 | 211 | 211 | 233 | 233 | 231 | 231 | 357 | 365 | 339 | 349 | 323 | 323 | MLG 61 | 3 |
| SA 115 | 315 | 318 | 286 | 286 | 237 | 253 | 288 | 288 | 214 | 214 | 224 | 233 | 231 | 231 | 365 | 365 | 339 | 339 | 308 | 308 | MLG 57 | 3 |
| SA 116 | 315 | 318 | 286 | 298 | 237 | 253 | 288 | 288 | 211 | 214 | 224 | 224 | 231 | 253 | 357 | 357 | 339 | 339 | 308 | 308 | MLG 36 | 5 |
| SA 117 | 315 | 318 | 286 | 298 | 237 | 253 | 274 | 274 | 214 | 214 | 224 | 233 | 231 | 253 | 357 | 365 | 339 | 349 | 308 | 323 | MLG 3 | 8 |
| SA 119 | 315 | 318 | 286 | 286 | 253 | 253 | 274 | 288 | 211 | 211 | 224 | 224 | 231 | 253 | 357 | 357 | 349 | 349 | 323 | 323 | MLG 58 | 3 |
| SA 120 | 315 | 318 | 286 | 286 | 237 | 253 | 274 | 288 | 214 | 214 | 224 | 233 | 253 | 253 | 357 | 365 | 339 | 349 | 308 | 323 | MLG 10 | 7 |
| SA 121 | 315 | 318 | 286 | 298 | 237 | 253 | 274 | 288 | 214 | 214 | 224 | 224 | 231 | 231 | 365 | 365 | 339 | 349 | 308 | 323 | MLG 12 | 6 |
| SA 122 | 318 | 318 | 286 | 298 | 237 | 253 | 274 | 288 | 211 | 214 | 224 | 224 | 253 | 253 | 365 | 365 | 339 | 349 | 323 | 323 | MLG 29 | 5 |
| SA 123 | 315 | 315 | 286 | 298 | 253 | 253 | 274 | 288 | 211 | 214 | 224 | 233 | 253 | 253 | 357 | 365 | 339 | 349 | 308 | 323 | MLG 7 | 7 |
| SA 125 | 315 | 318 | 286 | 298 | 237 | 253 | 274 | 288 | 211 | 214 | 224 | 233 | 231 | 253 | 357 | 365 | 339 | 349 | 308 | 323 | MLG 1 | 10 |
| SA 127 | 315 | 318 | 286 | 286 | 253 | 253 | 274 | 288 | 214 | 214 | 233 | 233 | 231 | 253 | 357 | 365 | 339 | 339 | 308 | 308 | MLG 44 | 4 |
| SA 128 | 315 | 315 | 286 | 298 | 237 | 237 | 274 | 274 | 214 | 214 | 233 | 233 | 231 | 253 | 365 | 365 | 339 | 349 | 308 | 323 | MLG 49 | 4 |
| SA 131 | 318 | 318 | 298 | 298 | 237 | 253 | 274 | 288 | 211 | 211 | 233 | 233 | 231 | 253 | 357 | 357 | 339 | 349 | 308 | 308 | MLG 48 | 4 |
| SA 132 | 315 | 315 | 286 | 298 | 237 | 237 | 274 | 274 | 214 | 214 | 233 | 233 | 231 | 253 | 365 | 365 | 339 | 349 | 308 | 323 | MLG 49 | 4 |
| SA 133 | 315 | 315 | 286 | 298 | 237 | 237 | 274 | 274 | 214 | 214 | 233 | 233 | 231 | 253 | 365 | 365 | 339 | 349 | 308 | 323 | MLG 49 | 4 |
| SA 134 | 315 | 318 | 286 | 298 | 237 | 253 | 274 | 288 | 214 | 214 | 233 | 233 | 253 | 253 | 357 | 357 | 339 | 349 | 308 | 323 | MLG 13 | 6 |
| SA 135 | 315 | 318 | 286 | 286 | 237 | 253 | 274 | 288 | 211 | 214 | 233 | 233 | 253 | 253 | 357 | 365 | 339 | 339 | NB | NB | MLG 31 | 5 |
| SA 136 | 315 | 315 | 286 | 298 | 237 | 237 | 274 | 274 | 214 | 214 | 224 | 233 | 231 | 253 | 365 | 365 | 339 | 349 | 308 | 323 | MLG 23 | 5 |
| SA 137 | 318 | 318 | 286 | 298 | 237 | 253 | 274 | 288 | 211 | 211 | NB | NB | 231 | 253 | 357 | 357 | 339 | 349 | 308 | 308 | MLG 27 | 5 |
| SA 138 | 315 | 318 | 286 | 298 | 237 | 253 | 274 | 274 | 211 | 214 | 224 | 233 | 231 | 253 | 357 | 365 | 339 | 349 | 308 | 323 | MLG 2 | 9 |
| SA 139 | 315 | 318 | 298 | 298 | 253 | 253 | 274 | 288 | 211 | 214 | 233 | 233 | 253 | 253 | 357 | 365 | 339 | 339 | 308 | 323 | MLG 33 | 5 |
| SA 141 | 315 | 318 | 286 | 298 | 237 | 237 | 288 | 288 | 211 | 211 | 224 | 224 | 231 | 253 | 357 | 357 | 339 | 349 | 308 | 323 | MLG 35 | 5 |
| SA 142 | 315 | 318 | 286 | 298 | 237 | 253 | 274 | 274 | 211 | 211 | 233 | 233 | 231 | 231 | 357 | 357 | 339 | 349 | 308 | 323 | MLG 34 | 5 |
| SA 144 | 315 | 315 | 286 | 298 | 253 | 253 | 288 | 288 | 214 | 214 | 233 | 233 | 231 | 253 | 357 | 365 | 339 | 349 | 308 | 323 | MLG 24 | 5 |
| SA 146 | 315 | 318 | 298 | 298 | 253 | 253 | 274 | 288 | 211 | 214 | 233 | 233 | 253 | 253 | 357 | 365 | 339 | 339 | 308 | 323 | MLG 33 | 5 |
| SA 147 | 315 | 318 | 286 | 298 | 237 | 253 | 274 | 274 | 211 | 214 | 233 | 233 | 231 | 253 | 357 | 365 | 339 | 349 | 308 | 323 | MLG 5 | 8 |
| SA 148 | 315 | 318 | 298 | 298 | 237 | 253 | 274 | 288 | 211 | 214 | NB | NB | 253 | 253 | 357 | 365 | 339 | 339 | 308 | 323 | MLG 15 | 6 |
| SA 149 | 318 | 318 | 298 | 298 | 237 | 253 | 288 | 288 | 214 | 214 | 224 | 233 | 231 | 231 | 357 | 357 | 349 | 349 | 323 | 323 | MLG 63 | 2 |

^a^ The 10 SSR markers used are: M1 = RJ6N, M2 = RJ24, M3 = scaffold 821-34567, M4 = scaffold 750-153307, M5 = scaffold 962-172974, M6 = CPS09, M7 = SUNIPst 09-48, M8 = SUNIPst 10-06, M9= SUNIPst 11-21, and M10= SUNIPst 15-30. a1 = allele 1 and and a2 = allele 2. SA = single-aeciospore isolate. NB = no band.
